# Update of Atomic Data for the First Three Spectra of Actinium

**DOI:** 10.3390/atoms10020042

**Published:** 2022-04-22

**Authors:** Alexander Kramida

**Affiliations:** National Institute of Standards and Technology, Gaithersburg, MD 20899, USA;

**Keywords:** atomic databases, standard reference databases, atomic spectroscopy, actinides, actinium, spectral lines, energy levels, transition probabilities, ionization energy

## Abstract

The present article describes a complete reanalysis of all published data on observed spectral lines and energy levels of the first three spectra of actinium (Ac I–III). In Ac I, three previously determined energy levels have been rejected, 12 new energy levels have been found; for six previously known levels, either the *J* values or the energies have been revised, and the ionization energy has been redetermined with an improved accuracy. In the line list of Ac I, three previous classifications have been discarded, 16 new ones have been found, and three have been revised. In Ac II, 16 new energy levels have been established, and 36 new identifications have been found for previously observed but unclassified lines. In both Ac I and Ac II, new sets of transition probabilities have been calculated. For all three spectra, complete datasets of critically evaluated energy levels, observed lines, and transition probabilities have been constructed to serve as recommended data on these spectra.

## 1. Introduction

Strange as it may seem, the spectra of actinide atoms and ions are important for astrophysics, as all radioactive elements with atomic numbers *Z* = 84 through 99, except for At (*Z* = 85) and Fr (*Z* = 87), have been detected in chemically peculiar stars (see a good review of these observations in Quinet et al. [1]. In addition to that, spectra of atomic and ionized actinium have many important applications. The isotope ^225^Ac is used in cancer radiotherapy, while ^227^Ac is usable in radioisotope thermoelectric generators, neutron radiography, tomography, and other radiochemical investigations, as well as serving as a tracer for deep seawater circulation and mixing (see Zhang et al. [2] and references therein). Knowledge of the spectrum of atomic Ac helps in developing efficient laser-ionization methods for isotope separation. It also has potential applications in studies of atomic parity and time-reversal violation [3]. Spectra of Ac^+^ and Ac^2+^, along with other actinide ions, have good prospects in the search for variation of the fine-structure constant [4]. Ac^2+^ is also of interest in parity nonconservation research [5]. Thus, it is not surprising that many tens of papers have been published on these spectra. Comprehensive lists of these publications can be found in the bibliographic databases of National Institute of Standards and Technology (NIST) on atomic energy levels [6] and transition probabilities [7] accompanying the NIST Atomic Spectra Database (ASD) [8].

Despite the high interest in the spectra of actinium, the information on its spectra in the NIST ASD is rather scarce, and their theoretical interpretation is incomplete. Most of it is based on the comprehensive experimental work of Meggers et al. [9] on the first three actinium spectra (Ac I–III), which was a result of several years of work and was published in 1957. Meggers et al. have produced many tens of high-resolution spectrograms recorded on photographic plates archived at NIST. Since then, only a few fragmentary observations have been made using laser spectroscopy methods. Atomic theory has also made little progress in interpretation of these spectra. In 2020, the team supporting the NIST ASD [8] has critically evaluated all presently available data on these spectra and prepared updated datasets of energy levels and spectral lines for Ac I–III. The original purpose of the present work was to document these updated datasets. However, in the course of the work, new information on Ac I was obtained, which indicated that much of the original analysis that led to the 2020 revision of ASD was incorrect. This led to a complete reanalysis of both the Ac I and Ac II spectra, which is described here. The analysis of Ac III was found to be correct, and it is also described here.

## 2. Data of Meggers et al.

As described in Meggers et al. [9], those authors had only a few mg of actinium to conduct their spectral investigation. With that, they recorded about 150 photographic plates with spectrograms obtained with two different grating spectrographs, as well as a few Fabry–Perot spectrograms for analysis of hyperfine structure. Most of the spectrograms were taken with a hollow cathode light source, which produces mostly neutral-atom spectra. Several spectrograms were also taken with copper and silver spark discharges, where Ac II and especially Ac III lines were strongly enhanced. Still, the wavelength range was restricted to (2000–11,000) Å to target mostly the Ac I and Ac II spectra. The grating used in an initial study was blazed at 6000 Å, but the best spectrograms were obtained with a grating blazed at 4000 Å, so the intensities of observed lines greatly decreased towards the ultraviolet and infrared ends of the spectrum. The main source of standard wavelengths used to calibrate the spectrograms was iron, the spectrum of which was photographed on the same plates using masking of portions of the entrance slit of the spectrograph or a movable mask near the photographic plate. Auxiliary standards were also supplied by numerous impurities present in the light sources: boron, sodium, potassium, calcium, strontium, barium, magnesium, zinc, aluminum, silicon, iron, chromium, nickel, manganese, palladium, platinum, lanthanum, radium, and lead.

The wavelengths reported by Meggers et al. [9] are “the means of 2 to 13 measurements, except a few cases where the line was classified, although observed only once”. These mean wavelengths were converted to vacuum wavenumbers. Although the air dispersion formula used was not specified, the present analysis revealed that it was the formula from Edlén 1953 [10]. The wavelength uncertainties were not specified for each line; instead, a general statement was made: “The probable error in any wavelength is usually less than 0.01 Å; this is shown by consistent agreement of different measurements and by the close fit of classified lines.” Unfortunately, despite the high measurement precision, all wavelengths given by Meggers et al. [9] were rounded to two digits after the decimal point. However, in the tables of classified lines of Ac III, Ac II, and Ac I (Tables 3–7 of Meggers et al., respectively), the wavelengths are accompanied by wavenumbers, which are given with a greater relative precision, especially at longer wavelengths. In the present work, the wavenumbers of these classified lines were determined as weighted averages of the wavenumber values given in the tables and those obtained from the given air wavelengths with the air dispersion formula mentioned above. Then these mean wavenumbers were converted to air wavelengths using the now-standard five-parameter formula of Peck and Reeder [11]. Thus, the missing third digit in the wavelength was approximately restored in about half of all wavelengths.

**Figure 1.**
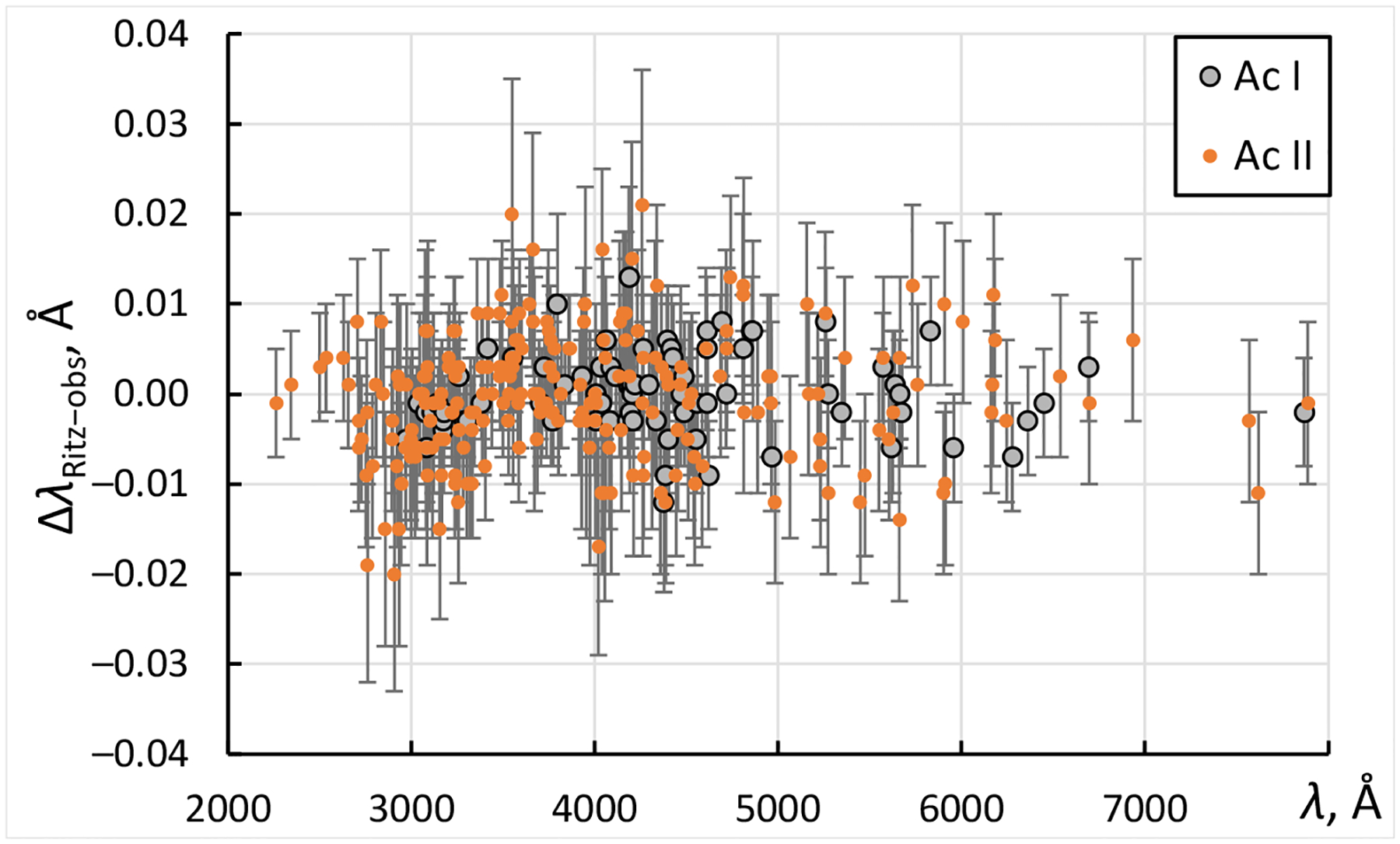
Observed wavelengths of Meggers et al. (1957) [9] compared with Ritz values. The error bars correspond to measurement uncertainties of Meggers et al. as assessed in the present work.

Since detailed information about uncertainties of observed wavelengths is not available, these uncertainties have been evaluated by comparison of observed and Ritz wavelength values, as described in Ref. [12]. Figure 1 shows a comparison of wavelengths observed by Meggers et al. [9] with Ritz wavelengths calculated from these observed wavelengths in a least-squares level optimization procedure (see below). Only the meaningful spectral lines are shown in this figure, i.e., those for which upper and lower levels of the transition are not defined by a single observed line.

A few classified lines of Ac III are not shown in this plot, but their consistency with Ritz values is similarly good. For Ac I, the root-mean-square (rms) values of the differences Δ*λ*_Ritz−obs_ plotted in Figure 1 are about 0.004 Å for wavelengths shorter than about 4000 Å and 0.005 Å for longer wavelengths. For Ac II, the corresponding rms values are 0.006 Å and 0.009 Å. The lines marked with characters in the intensity values (such as “h”—hazy line, “c”—complex line, etc.) showed a somewhat greater rms values, 0.006 Å for Ac I and 0.008 Å for Ac II. These estimates were adopted as measurement uncertainties for most lines. For a few lines showing greater deviations of observed wavelengths from the Ritz values, the uncertainties have been increased. For wavelengths of unclassified lines, which were all rounded to two decimal places after the point and were not accompanied by wavenumbers in the tables of Meggers et al., an uncertainty of 0.013 Å was adopted.

In addition to the first three spectra of actinium, Meggers et al. [9] have observed six lines tentatively assigned to Ac IV (no attempts have been made to classify these lines) and several bands of the AcO molecule.

## 3. Ac I

### 3.1. Revisions and Extensions of Experimental Data on Ac I

Considering the lack of good computational resources at the time Meggers et al. [9] carried out their work, the quality of their measurements and analysis is amazingly good. Nevertheless, they made some mistakes.

One such mistake is a wrong *J* = 3/2 value assigned to the level at 22,801.11 cm^−1^. This mistake was corrected by Ferrer et al. [13] (see also Granados et al. [14] and the earlier work of Sonnenschein [15]). These authors have analyzed the hyperfine structure (hfs) of the transition from this level to the ground level, which was observed by Meggers et al. [9] near 4384.5 Å (air wavelength). Their analysis of the observed hfs intervals unambiguously showed that this excited level has *J* = 5/2.

There are many other cases where the *J* values assigned by Meggers et al. [9] are not based on a unique choice allowed by observed combinations. A recent theoretical study by Dzuba et al. [3] suggested a number of possible revisions in those original *J* assignments. Some of them also involve a possible revision of excitation energy where the only observed transition could be associated not with the ground level 6d7s^2 2^D_3/2_ but with the first excited level with *J* = 5/2 of the same term.

On the other hand, the hfs study by Sonnenschein [15] has confirmed the *J*-values assigned by Meggers et al. [9] to three other low-excited levels at 25,729 cm^−1^ (*J* = 1/2), 26,066 cm^−1^ (*J* = 3/2), and 24,969 cm^−1^ (*J* = 7/2). For the latter level, the *J* = 7/2 assigned by Meggers et al. [9] is confirmed despite the fact that there is one line observed at 4003.79 Å (air wavelength; unclassified in the line list of Meggers et al. [9]) whose wave number (24,969.28 cm^−1^) almost exactly coincides with this level’s energy (24,969.294(17) cm^−1^, according to the present analysis). This line cannot be due to a transition from the 24,969 cm^−1^ level (*J* = 7/2) to the ground level (*J* = 3/2), as it is optically forbidden. Thus, the wave number coincidence mentioned above must be treated as fortuitous.

Since the study of Meggers et al. [9] was restricted to the wavelength range below 7887 Å, they could not observe transitions from the lowest odd-parity levels $\left(7\text{s}{\;}^{2}7\text{p}{\;}^{2}{\text{P}}_{1/2,3/2}^{{}^{\circ}}\right)$ to the levels of the ground term. These transitions were recently observed and identified by the Mainz laser spectroscopy group in collaboration with several other institutions (see Zhang et al. [2]). Among the wavelengths measured in that work, there is one line in common with Meggers et al. [9], which allows an independent check of the measurement accuracy. The wavenumber of the $6\text{d}7{\text{s}}^{2}{\;}^{2}{\text{D}}_{3/2}-6\text{d}7\text{s}\left({\;}^{3}\text{D}\right)7\text{p}{\;}^{4}{\text{F}}_{3/2}^{{}^{\circ}}$ transition was reported by Zhang et al. [2] as 13,712.74(3) cm^−1^, while it is 13,712.898(11) cm^−1^, as follows from the measurements of Meggers et al. The difference is 0.16(3) cm^−1^, corresponding to −0.084(16) Å. As seen from Figure 1, this difference is much greater than the measurement uncertainty of Meggers et al., which means that there was a significant unaccounted source of error in the measurements of Zhang et al. [2]. As privately communicated by some of those authors [16], the Mainz group continues their investigations of the lowest odd-parity levels of Ac I. They have remeasured these levels with high resolution, which allowed them to study in detail the hfs structure of the three levels reported by Zhang et al. [2] and confirm the energy 13,712.898(11) cm^−1^ of the $6\text{d}7\text{s}\left({\;}^{3}\text{D}\right)7\text{p}{\;}^{4}{\text{F}}_{3/2}^{{}^{\circ}}$ level following from the measurements of Meggers et al. [9]. The most probable cause of the error in the measurement of this level by Zhang et al. [2] is the large deviation of intensities of the hfs components of this level from those that assume theoretical line strengths and statistical populations of the hfs sublevels. Such large deviations were recently observed in other transitions of Ac I by Granados et al. [14], who used the same resonance ionization laser spectroscopy technique as Zhang et al. [2]. Granados et al. [14] gave detailed explanations for the reasons of these deviations. These deviations were ignored in the work of Zhang et al. [2], who assumed that the observed center of gravity of the transition corresponds to the difference between centers of gravity of the hfs structures of the two levels. On the other hand, the measurements of Zhang et al. [2] for the other two levels $\left(7\text{s}{\;}^{2}7\text{p}{\;}^{2}{\text{P}}_{1/2,3/2}^{{}^{\circ}}\right)$ have been confirmed by Raeder et al. [16].

Raeder et al. [16] have found that the transition observed at 4462.73 Å by Meggers et al. [9] originates not from the ground level, as classified by those authors, but from the metastable 6d7s^2 2^D_5/2_ level at 2231 cm^−1^. Its observed hfs structure indicates the *J*-value of the upper level to be 7/2.

Another finding of Raeder et al. [16] is that the levels at 23,475.94 cm^−1^ and 26,533.16 cm^−1^, which were assigned by Meggers et al. [9] to upper levels of transitions originating from the ground term 6d7s^2 2^D, are impossible to observe by resonance excitation-ionization laser spectroscopy technique employed by the Mainz group. Thus, these levels are likely to be spurious, despite the very good agreement of the wave number difference of the lines at 3767.800 Å and 4113.769 Å (2231.40(3) cm^−1^), which were assigned to the level at 26,533.16 cm^−1^ by Meggers et al. [9], with the splitting of the ground term (2231.432(8) cm^−1^, according to the present work).

Besides the determination of the three energy levels $(7\text{s}{\;}^{2}7\text{p}{\;}^{2}{\text{P}}_{1/2,3/2}^{{}^{\circ}}$ and $\text{6d}7\text{s}\left({\;}^{3}\text{D}\right)7{\;}^{4}{\text{F}}_{3/2}^{{}^{\circ}})$, the study of Zhang et al. [2] also includes a measurement of radiative lifetimes of these levels. They were found to be 668(11) ns, 255(7) ns, and 352(11) ns, respectively.

In addition to the studies discussed above, in 2012 Roßnagel et al. [17,18] experimentally determined the ionization energy (IE) of Ac I by analyzing three different Rydberg series in two-color resonant laser excitation. Their result, 43,394.45(19) cm^−1^, is presently adopted as the recommended value of the ionization energy of Ac I. With the current values of fundamental constants [19], it corresponds to 5.380226(24) eV. In their work, Roßnagel et al. assumed the measurements of Meggers et al. [9] to have rather large uncertainties, namely, 0.11 cm^−1^ for both the Ac I and Ac II levels used in their analysis. From the present data described below, these uncertainties are much smaller (0.02–0.03) cm^−1^, which calls for a repeated analysis of the measurements of Roßnagel et al. [17]. The value of the ionization energy can be improved with the present revised data of Meggers et al. [9]. This improvement is described below in Section 3.4.

All revisions and extensions discussed above have been incorporated in the present study and supplemented with several more new identifications. The complete line list with energy level classifications is presented in Table 1, and the list of energy levels (both the experimentally found and predicted ones) is given in Table 2 (both tables are placed at the bottom of this section to make reading of the text easier).

### 3.2. Theoretical Calculations for Ac I

The electronic structure of Ac I is very complex. The ground configuration is 6d7s^2^, which involves only one open shell, 6d. However, excited levels involve many overlapping and strongly interacting configurations with up to three open shells: 6d7s*nl*, 6d^2^*nl*, 7s^2^*nl*, and 7p^2^*nl*. Meggers et al. [9] based their analysis on the supposed analogy with homologous spectra, Sc I, Y I, and La I. However, relative positions and widths of the interacting configurations *n*′d(*n*′ + 1)s*nl*, *n*′d^2^*nl*, (*n*′ + 1)s^2^*nl*, and (*n*′ + 1)p^2^*nl* change with increasing *n*′ (*n*′ = 3 in Sc I, 4 in Y I, 5 in La I, and 6 in Ac I). This leads to redistribution of spectral line positions and strengths. Therefore, it is not surprising that many of the line classifications made by Meggers et al. [9] were erroneous. Luckily, the ground configuration of Ac I is still analogous to the homologous spectra, which simplified the initial analysis. The wavenumber difference of about 2231 cm^−1^ was seen in 20 pairs of observed lines and was readily identified as the fine-structure splitting within the ground term, 6d7s^2 2^D. As one can see in the Periodic Table of the Elements [20], analogy of the ground configurations with those of the lighter homologous elements does not hold for the first spectra of the neighboring elements Th, Pa, U, and Np, making their analysis much more difficult.

The most extensive and accurate calculation of spectroscopic properties of Ac I was made in the above-mentioned work of Dzuba et al. [3]. This work used a combination of the configuration interaction and the linearized single-double–coupled-cluster methods (CI + SD). As seen from Table I of Dzuba et al., for levels below about 20,000 cm^−1^, their calculated energies agree with experimental values of Meggers et al. [9] within a few hundred cm^−1^. For higher energies, the discrepancies increase by an order of magnitude, and there are many ambiguities in association of the calculated levels with experimental ones. Dzuba et al. pointed out that many of the experimental energies could be wrong when they are determined by a single observed line. In those cases, the lower level of the corresponding transitions may differ from the interpretation of Meggers et al. There are also many cases where the *J* values assigned by Meggers et al. may be in error.

This illustrates the old problem: How do we establish a correspondence between the theoretical energy structure and that observed in experiments? A similar problem also occurs in comparisons of different theoretical models with each other. As explained by Kramida [12], the best method is to use the patterns of calculated transition probabilities from each level. In comparisons with experiments, these patterns should be matched with patterns of observed line intensities. This avoids the problem of unknown distributions of level populations in experimental spectra, since only the branching ratios are involved in the comparison. However, it requires the spectral variations of the registration sensitivity to be removed from the observed intensities, and relies on the assumption that the plasma is optically thin in the light source used. For establishing a correspondence between different theoretical models, the patterns of calculated transition probabilities can be directly compared. Energy level associations derived by this technique are much more reliable than associations based on energy ordering.

**Figure 2.**
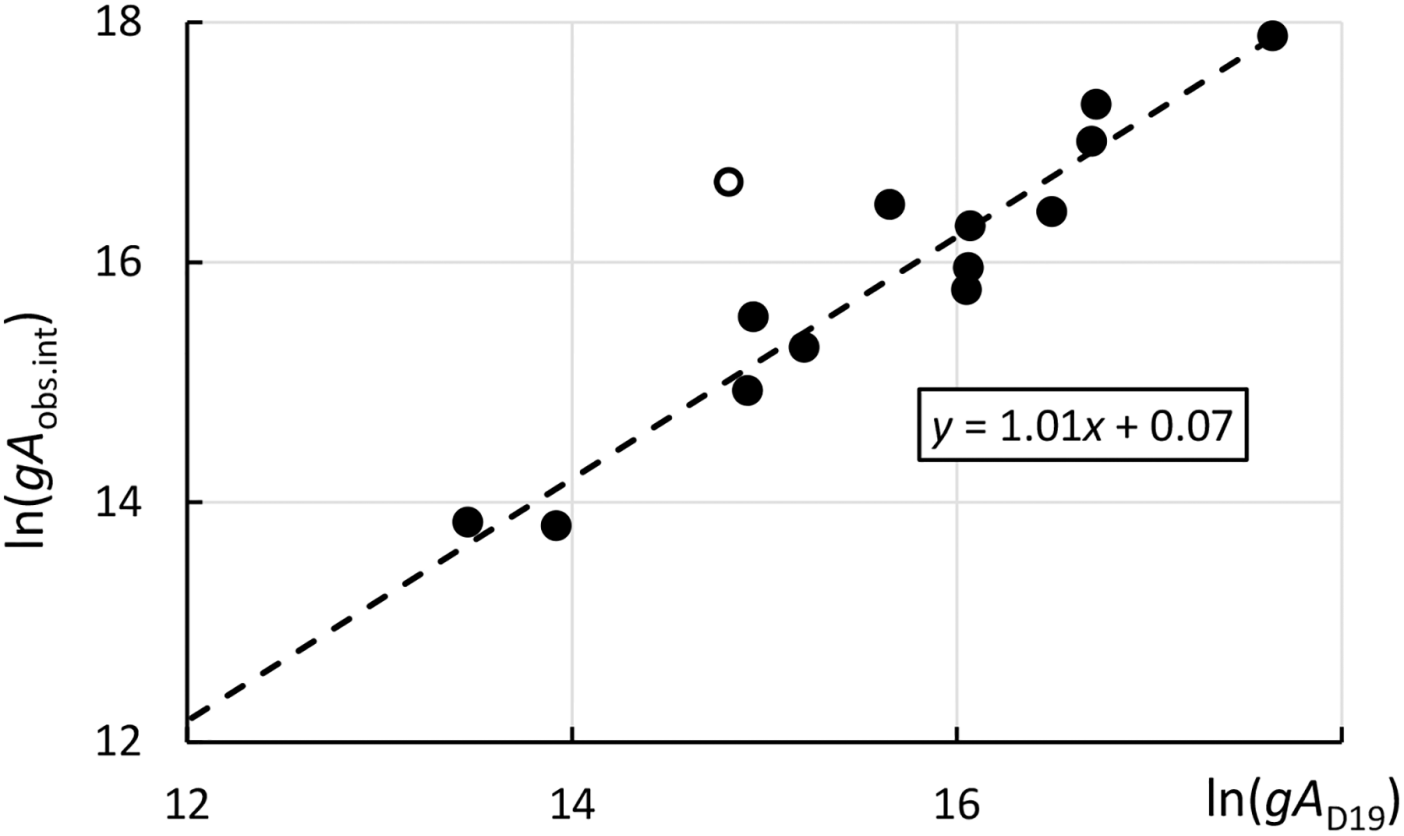
Comparison of observed line intensities with calculated transition rates (*gA* values) of Dzuba et al. [3]. The quantity *gA*_obs.int._ on the vertical axis represents the *gA* values derived from observed intensities reduced in the present work to a common linear scale (see Section 6). The empty circle represents the line at 5228.31 Å tentatively identified in this work as the transition from the $6\text{d}7\text{s}\left({\;}^{3}\text{D}\right)7\text{p}{\;}^{4}{\text{D}}_{5/2}^{{}^{\circ}}$ level to the ground level (see the text). This transition was excluded from the linear fit depicted by the dashed line.

Dzuba et al. [3] provided their calculated transition probabilities for 66 lines originating from seven odd-parity levels. Intensities of the lines originating from these levels observed in experiments are compared in Figure 2 with those calculated by Dzuba et al. [3].

The observed intensities used in this figure are the values that have been reduced in the present work to a common scale (see Section 6). The quantity *gA*_obs.int._ plotted in the figure is directly proportional to these intensities:

(1) $$gA_{\text{obs.int. }}=\frac{I_{\text{obs }}\lambda }{\exp\left(-E_{\text{up }}/0.5121+3.693\right)},$$

where *I*_obs_ is the observed intensity (in arbitrary units on the scale adopted here; see Section 6), *λ* is transition wavelength in angstroms, and *E*_up_ is the energy of the upper level in eV.

As one can see from Figure 2, agreement of the calculated *gA* values of Dzuba et al. [3] with observed intensities is very good (except for one tentatively identified line, which will be discussed further below in Section 3.3). Even more impressive is the agreement of the calculated lifetimes with the three experimental values of Zhang et al. [2]. After the *A*-values reported by Dzuba et al. [3] have been adjusted to experimental transition energies, the lifetimes calculated from these adjusted *A*-values agree with experiment within 10% on average. This estimate coincides with uncertainties given by Dzuba et al. [3] for their calculated lifetimes.

The other three published datasets of energy levels and transition rates for Ac I are those of Quinet et al. (2007) [1], Özdemir and Ürer (2010) [23], and Ürer and Özdemir (2012) [24]. Compared to the calculations of Dzuba et al. [3], those older ones are all of a relatively small scale. While all three older calculations account for only a limited amount of valence–valence electron correlations (by inclusion of 23/25 [1], 24/23 [23], and 13/5 [24] valence-excited configurations of even/odd parity), those of Dzuba et al. [3] effectively accounted for core–valence correlations (in the SD part of their method) and included a few hundred thousands of configurations in the CI part of their calculation. Consequently, the results of those older calculations are of very limited accuracy.

Calculations of Quinet et al. [1] were made with the pseudo-relativistic Hartree–Fock method with inclusion of core polarization (HFR+CPOL) implemented in a modified version of Cowan’s atomic structure codes [21]. Addition of the core-polarization potential effectively accounts for core–valence interactions, which could potentially give reasonable results. Semiempirical adjustments were made to the average energies of configurations and spin–orbit interaction parameters in a least-squares fitting (LSF) of experimentally known energy levels. However, as noted by Quinet et al. [1], the odd-parity energy levels of Meggers et al. [9] could not be fitted with a reasonable accuracy. As discussed in the previous subsection, one of the reasons for that is presence of several incorrectly identified levels in Ref. [9].

In the present work, an attempt was made to calculate the Ac I spectrum with another modification of Cowan’s codes [22]. As in the work of Quinet et al. [1], no configurations involving excitation from the Rn-like [Hg]6p^6^ core were considered. The following configurations were included in these calculations: in even parity, [Rn]6d(7s^2^ + 7p^2^ + 8s^2^ + 7s5g + 7s7d + 7s8s + 7p8p + 7p5f + 8p5f + 7d8s), 7s(7p^2^ + 8s^2^ + 7p5f + 7p8p + 8p5f + 7d8s) + 6d^2^(7s + 7d + 8s + 5g) + 6d^3^ + 7s^2^(5g + 8s + 7d) + 7p^2^(8s + 7d) + 7p8s(8p + 5f) + 7p7d5f + 8s8p5f (30 configurations in total); in odd parity, [Rn]6d7s(7p + 8p + 5f) + 6d7p(8s + 7d + 5g) + 6d8s(8p + 5f) + 6d^2^(7p + 8p + 5f) + 6d7d(8p + 5f) + 7s7d(8p + 5f) + 7s^2^(7p + 8p + 5f) + 8s^2^(7p + 8p) + 7p^2^(8p + 5f) + 7s7p(8s + 7d + 5g) + 7s8s(8p + 5f) + 7p^3^ + 7p8p5f (29 configurations in total). These configuration sets are larger than those used in the study of Quinet et al. [1] (23 and 25 configurations in the even- and odd-parity sets, respectively). It should be noted that the present extension of the configuration sets included in the calculation does not replace the core-polarization corrections that were accounted for in the calculations of Quinet et al. [1], because no core-excited configurations were included here. Nevertheless, in the case of Ac I, a better account for interactions between the valence-excited configurations proved to be much more important than the effects of core excitations.

The calculation started with an attempt to reproduce the theoretical levels of Dzuba et al. [3] by adjusting the Slater parameters in a LSF. The immediate problem turned out to be with level designations of Dzuba et al. [3]. They did not calculate the eigenvector compositions. Instead, their level labels were assigned by using an ad hoc procedure involving a search for a combination of *L* and *S* quantum numbers that would give the best match between the Landé *g*-factor returned by a simple formula assuming pure *LS* coupling and the *g*-factor calculated by Dzuba et al. [3] with the CI + SD method. This method of labeling often results in unphysical term designations. For example, the level list of Dzuba et al. includes two 6d^3 4^D_3/2_ levels, one at 28,793 cm^−1^ and another at 34,409 cm^−1^. Both designations are invalid, because there is no ^4^D term in the 6d^3^ configuration (see, e.g., Martin et al. [25]). Configuration labels are also ambiguous because of strong CI (note that the level predicted at 33,551 cm^−1^ with *J* = 3/2 was wrongly designated by Dzuba et al. [3] as 7s^2^9p. Most probably, it was just a misprint: it must be 7s^2^8p, as follows from the present calculations). Thus, we are left with only two options available for matching the level structure calculated by Dzuba et al. [3] with that returned by Cowan’s codes: (1) use the patterns of calculated transition rates (given by Dzuba et al. [3] for only a few lowest excited levels) and (2) use the Landé factors to identify the levels. By using these two methods, it turned out to be possible to make a reasonably good fit. It should be significantly better than an ab initio calculation with Cowan’s codes, since this procedure effectively introduces corrections to Slater parameters, partially accounting for configuration interactions and relativistic effects missing in the ab initio Cowan-code calculation.

The LSF calculation then proceeded with replacement of theoretical levels of Dzuba et al. [3] with experimental values, where the identification was deemed reliable, and comparing the resulting predicted line intensities with the observed ones. Several sets of LSF calculations were made. In each set, all predicted levels and strong transitions were loaded into the input files for the visual line identification code IDEN2 [26], and the patterns of predicted intensities were compared with those present in the experimental line list of Meggers et al. [9]. If a level was found to be wrongly assigned to a set of observed lines (i.e., the observed intensities did not match the predicted ones for this level), or if a new, previously unknown level was found, the LSF was repeated with a corrected or expanded set of experimental levels, and a new session of work with IDEN2 was initiated. In total, several tens of LSF calculations were made, gradually extending the list of established levels and improving the match between observed and calculated line intensities.

For many odd-parity levels above 20,000 cm^−1^, the calculated *A*-values were found to be very sensitive to small changes of Slater parameters. This is due to strong interactions between the configurations involved. It indicated that, to reliably predict the *A*-values, the positions of all strongly interacting configurations must be established with an error not exceeding a few hundred cm^−1^. Fortunately, the calculations of Dzuba et al. [3] have provided enough sufficiently accurate data to make it possible.

**Figure 3.**
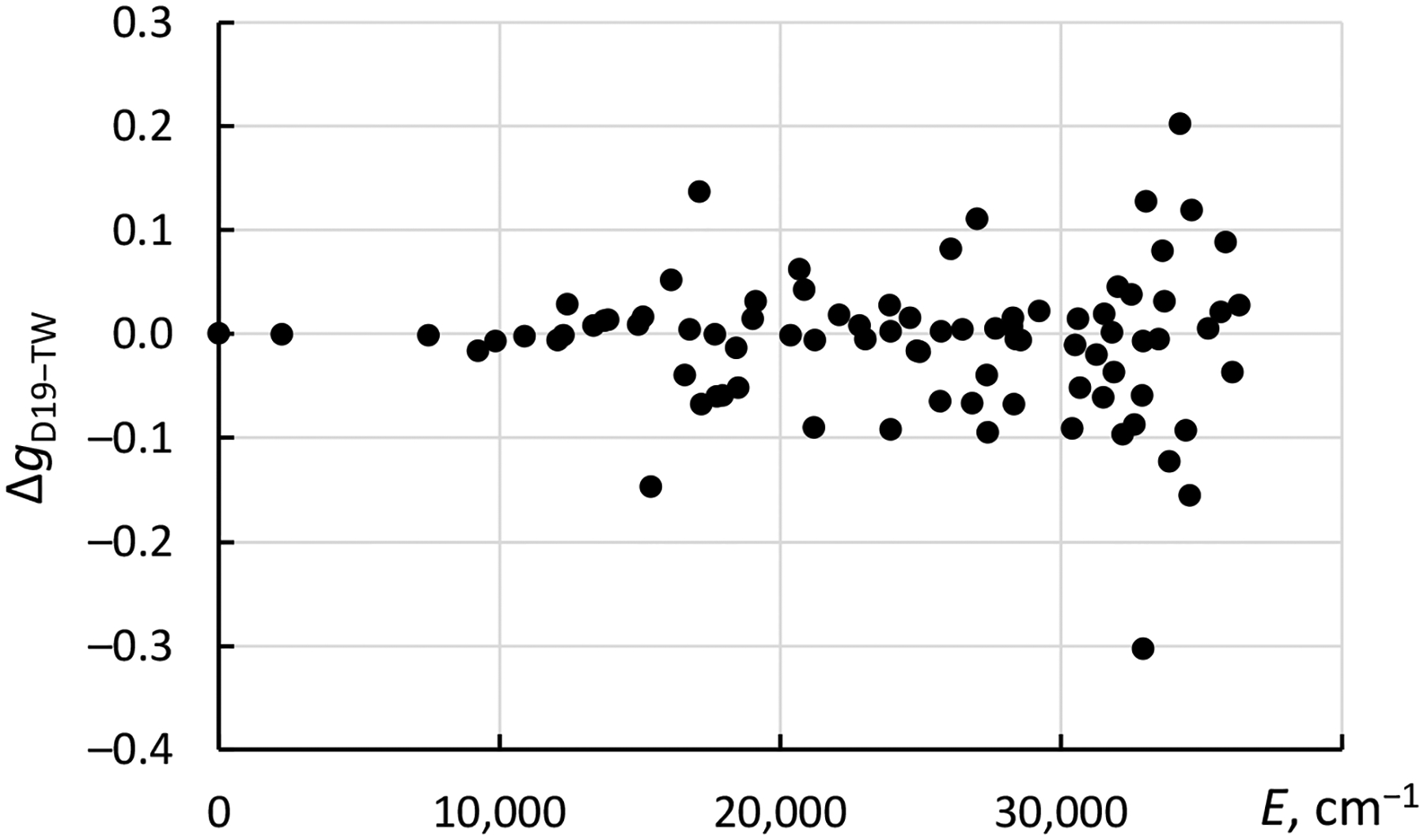
Differences of Landé *g*-factors calculated by Dzuba et al. [3] from those computed in the present work in a least-squares fitting with Cowan’s codes.

In the end, a rather good agreement between the results of the present calculations with those of Dzuba et al. [3] has been achieved. The Landé *g*-factors calculated in the present LSF with Cowan’s codes are compared with those of Dzuba et al. [3] in Figure 3.

As this figure shows, agreement between the two calculations is very good for the low-excited levels below about 15.1 kK (1 kK = 1000 cm^−1^). The rms difference between the two sets of calculated *g*-values is 0.012 for these low-excited levels. For higher levels with energies between 15.1 kK and 32.9 kK, the rms difference is 0.05. For the levels above 32.9 kK, it grows to 0.12. In the absence of any experimental data for comparison, these estimates can be adopted as uncertainties for both sets of calculated data.

**Figure 4.**
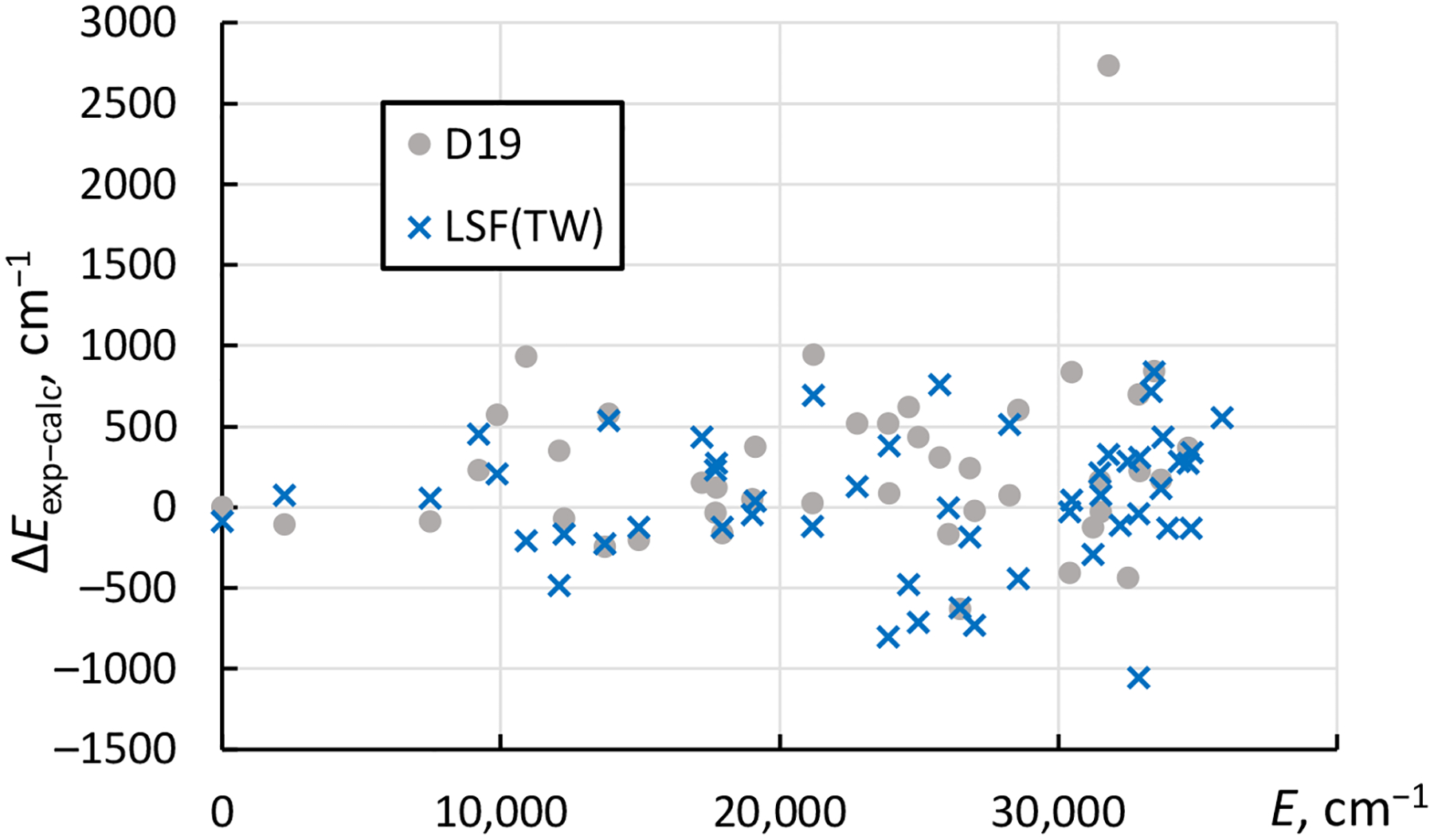
Differences of energy levels calculated by Dzuba et al. [3] (D19) and in the present LSF calculation (TW) from experimental values.

A similar pattern is seen in comparison of the calculated energy levels with observed ones, shown in Figure 4.

For the even-parity levels, the rms difference of the presently calculated levels from experimental ones is 419 cm^−1^, while for the calculation of Dzuba et al. [3] it is 531 cm^−1^. For the odd-parity levels, the corresponding rms values are 411 cm^−1^ and 607 cm^−1^. The one level that shows an outstandingly large difference between the calculation of Dzuba et al. [3] and experiment is $5\text{f}7{\text{s}}^{2}{\;}^{2}{\text{F}}_{5/2}^{{}^{\circ}}$. It is interesting to note that for the other 5f7s^2 2^F° level with *J* = 7/2, the result of Dzuba et al. [3] is in rather good agreement both with the present calculation and with the experimental value. Both these levels are almost pure in *LS* coupling (with more than 90% of the leading term in their eigenvector compositions). The cause of the discrepancy in the calculation of Dzuba et al. is unclear.

The calculated level values, their eigenvector compositions, and Landé factors found in the present LSF calculations are included in the level list given in Table 2 together with experimental energies and calculated radiative lifetimes (the latter are described in the following section). The final fitted values of the Slater parameters resulting from the present LSF are listed in Table 3.

### 3.3. Ac I Transition Probabilities

As mentioned in Section 3.1, radiative lifetimes of the lowest three odd-parity levels of Ac I were measured by Zhang et al. [2]. The measured lifetime of the $7\text{s}{\;}^{2}7\text{p}{\;}^{2}{\text{P}}_{1/2}^{{}^{\circ}}$ level directly gives the $6\text{d}7{\text{s}}^{2}{\;}^{2}{\text{D}}_{3/2}-7\text{s}{\;}^{2}7\text{p}{\;}^{2}{\text{P}}_{1/2}^{{}^{\circ}}$ transition probability, as this is the only allowed channel of radiative decay of this level. For the two allowed electric dipole (E1) transitions from the $7\text{s}{\;}^{2}7\text{p}{\;}^{2}{\text{P}}_{3/2}^{{}^{\circ}}$ level, as well as for two E1 transitions from the $6\text{d}7\text{s}\left({\;}^{3}\text{D}\right)7\text{p}{\;}^{4}{\text{F}}_{3/2}^{{}^{\circ}}$ level, the present recommended values of transition probabilities (*A*-values) have been derived from the branching fractions computed by Dzuba et al. [3] combined with the lifetimes measured by Zhang et al. [2]. The five experimental and semiempirical *A*-values described above have been complemented by five *A*-values computed ab initio by Dzuba et al. [3], which have been assigned accuracy categories C+ (one transition), C (one transition), D+ (two transitions), and E (one transition). These accuracy categories correspond to uncertainties ≤18%, ≤25%, and ≤40%, and >50%, respectively. In addition to that, 85 *A*-values calculated in the present work (with Cowan’s codes [22]) have been adopted. This selection was based on comparison of the present calculation with that of Dzuba et al. [3], as well as with observed line intensities. Eight of the presently computed *A*-values are for parity-forbidden transitions, which will be discussed further below. Of the 77 presently computed *A*-values of E1 transitions, 38 have been assigned to the accuracy category B (uncertainties ≤10%), 20 to the accuracy categories C, D+, and D (uncertainties ≤25%, ≤40%, and ≤50%), while the remaining 19 are estimated to be accurate only to a factor of about two, corresponding to the accuracy category E.

**Figure 5.**
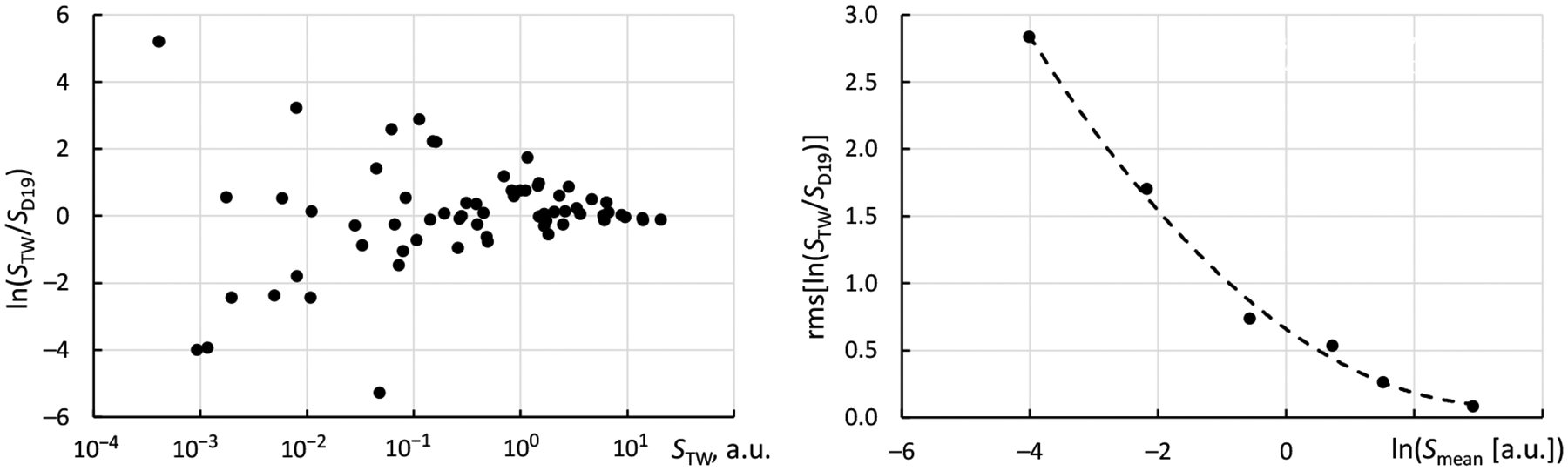
**Left** panel: Comparison of transition line strengths *S* (in atomic units, a.u.) calculated by Dzuba et al. [3] (D19) and in the present work (TW). **Right** panel: Derivation of the uncertainty estimate (see the text).

In the present calculation of allowed (E1) transitions, the reduced E1 transition matrix elements calculated by Cowan’s codes [21,22] were reduced for s–p and p–d transitions by a factor of 0.811 to bring the calculated lifetimes of the lowest odd-parity levels in agreement with those observed by Zhang et al. [2] and calculated by Dzuba et al. [3] for Ac I. For the d–f and f–g transitions, the scaling factor was set at 0.8233 as determined in the analysis of Ac III data (see Section 5). The comparison of the presently calculated line strengths *S* with those calculated by Dzuba et al. [3] is depicted in Figure 5.

As seen in the left panel of Figure 5, the comparison shows a typical pattern of very small differences between the two calculations for the strongest transitions, while the agreement rapidly deteriorates with decreasing line strength *S*. To obtain numerical estimates of uncertainties as a function of *S*, following the method suggested by Kramida [12], the entire range of *S*-values available for comparison was divided into six intervals divided by the following values of *S* computed in this work (*S*_TW_): 6.7 a.u., 3.0 a.u., 1.3 a.u., 0.18 a.u., and 0.06 a.u. In the right panel of Figure 5, the rms values of the natural logarithm of the ratio *S*_TW_/*S*_TW_ are plotted against the average values of *S*_TW_ available for comparison in each interval. The quadratic interpolation depicted by the dashed line was then used to produce an estimate of uncertainty (in *S*) of the present calculation as a function of *S*.

As mentioned in the previous subsection, the earlier calculations of Refs. [1,23,24] were too inaccurate to be usable in such comparisons. Thus, in the absence of a better benchmark, the same function of *S* as described above was used to estimate the uncertainties of the calculation of Dzuba et al. [3]. For a few of the strongest transitions, even though the estimated uncertainties are similar, the data of Ref. [3] were given a higher preference. For weaker transitions, a comparison of calculated and observed line intensities was used as an additional selection tool. A few of the presently calculated *A*-values were found to better agree with the observations, so they were selected as recommended values.

In addition to E1 transitions, the *A*-values for the parity-forbidden magnetic dipole (M1) and electric–quadrupole (E2) transitions were calculated in the present work. No data on these transitions are currently available in the literature. Therefore, no comparisons with other data are available for estimation of uncertainties of these results. Thus, the Monte Carlo method suggested by Kramida [27] was used for this estimation. One hundred random trial calculations were made with the Slater parameters, and E2 transition matrix elements varied around their nominal values within the normal statistical distributions. The widths of these distributions for the Slater parameters were set by the standard deviations of the LSF, while for the E2 transition matrix elements, a variance of 15% was assumed. A transition was deemed to be of a “mixed” M1 + E2 type if the contribution of the E2 transition to the total *A*-value was found to be 2% or greater. A total of eight forbidden transitions are included in Table 1. Four of them are M1 transitions, for which the calculated *A*-values are very accurate (category A+ or AA). The accuracy is much worse for the E2 transitions and for the mixed-type transitions having a large E2 contribution. However, for the selected four transitions of this kind, the accuracy category is C+ or C (uncertainties ≤25%).

The radiative lifetimes included in Table 2 were calculated by summing up the *A*-values for all radiative decay branches of each level, including the E1, M1, and E2 transitions. Their uncertainties were calculated by a standard statistical formula for propagation of uncertainties of each participating *A*-value. Where the reference lifetime values are available experimentally [2] or theoretically [3], the present values agree with the reference values within 14% on average.

### 3.4. Ionization Energy of Ac I

As mentioned in Section 3.1, the ionization energy (IE) of Ac I was determined by Roßnagel et al. [17] to be 43,394.45(19) cm^−1^. In that determination, those authors adopted the values of intermediate excited levels of Ac I from Meggers et al. [9] and assumed their uncertainties to be 0.11 cm^−1^. The same assumption was made about the excitation energies of Ac II levels used to calculate the limit offsets for the Rydberg series converging to excited levels of Ac II. In the present work, the values of all these levels have been significantly refined. These new values are in agreement with those of [9], but their uncertainties are found to be much smaller, between 0.02 cm^−1^ and 0.03 cm^−1^. Thus, a new determination of the IE was made here by using the data for the same Rydberg series as used by Roßnagel et al. [17]. The original measured wave numbers of the observed lines were taken from the master thesis of Roßnagel [18]. The IE was determined by a least-squares fit of the extended Ritz-type quantum-defect expansion formula (see, e.g., Ref. [12]). A recently written (by the present author) computer code *fit_Ritz*, which simultaneously fits multiple Rydberg series with a common IE value, was used. Unlike the multi-channel quantum defect formulas used by Roßnagel et al. [17], the formula used here cannot account for perturbations caused by configuration interactions. Therefore, the perturbed members of the series labeled as b and c in Roßnagel et al. [17], which converge to excited levels of Ac II, have been excluded from the fit. Namely, the levels with *n* = 16 to 25, 33, 36, and 46 have been excluded from the series b converging to the 6d7s ^3^D_1_ level of Ac II at 4739.631(33) cm^−1^ (see Section 5). From the series c, converging to the 6d7s ^3^D_2_ level of Ac II at 5267.147(32) cm^−1^, levels with *n* = 24 to 34, 41, 47, 48, and 49 have been excluded. In the multi-channel quantum defect fit of Roßnagel et al. [17], the additional free variables pertaining to the perturbing levels have largely absorbed the additional degrees of freedom corresponding to the levels excluded here. Thus, these exclusions are not expected to signif icantly deteriorate the accuracy of the fit for the IE. Indeed, when each of the series b and c are fitted separately, their limits are found to be 43,394.39(6) cm^−1^ and 43,394.98(39) cm^−1^, respectively, where the uncertainties are purely statistical. The corresponding values from Roßnagel et al. [17] are 43,394.25(27) cm^−1^ and 43,394.59(43) cm^−1^. The present results agree with those of Roßnagel et al. [17], and the statistical uncertainties are even smaller. The most precise determination of the IE is provided by the unperturbed series a, for which 36 members were observed by Roßnagel [18]. The series limit derived here from this single series is 43,394.524(21) cm^−1^. This agrees with the value of Roßnagel et al. [17] obtained for this series, 43,394.530(3) cm^−1^. Here, the uncertainties are again purely statistical. However, it is evident that the meaning of “statistical” is different in the present work and in Roßnagel et al. [17]. The greater uncertainty of the present value stems from inclusion of the systematic part of the wavelength-measurement uncertainty (0.04 cm^−1^, according to Raeder et al. [16]) in the uncertainties of the measured wave numbers, which are the input values for the present fit. Here, this systematic error was assumed to vary quasi-randomly in measurements of the different peaks. It makes little difference for the determination of the total uncertainty, since other systematic effects are estimated to be significantly larger. The most significant systematic error stems from the asymmetry of the observed peaks caused by unresolved fine and hyperfine structure. The largest of these errors is due to the hfs of the lower level, from which the Rydberg levels were excited. For the series a, it was the level at 31,800.350(18) cm^−1^, which is presently identified as $5\text{f}7{\text{s}}^{2}{\;}^{2}{\text{F}}_{5/2}^{{}^{\circ}}$ (note that in the work of the Mainz group [17,18], this level was assumed to have *J* = 3/2, following Meggers et al. [9]). The possible effect of the asymmetry of this level caused by hfs is estimated to be about 0.1 cm^−1^. This systematic error dominates the uncertainty of the final result of the present joint fit of the three series (a, b, and c), which is 43,394.52(10) cm^−1^. With the present values of the fundamental constants [19], it corresponds to 5.380235(13) eV. This value agrees with the determination of Roßnagel et al. [17] but is about twice more precise.

### 3.5. Ac I: Discussion and Outlook

The experimental values of the energy levels given in Table 2 have been determined from the wavelengths of identified lines by the least-squares optimization procedure using the code LOPT [28]. Their uncertainties are estimated as the maximum of two values returned by LOPT, *D*_1_, and *D*_2_ (for definitions of these quantities, see Ref. [28]). This code also determines the uncertainties of the Ritz wavelengths, which include the covariances between the optimized values of the lower and upper levels of each transition. These uncertainties are also given in Table 2.

As can be seen in the last column of Table 2, the present level list includes 13 newly identified levels (eight of which are firmly established, and five are tentatively identified based on one observed line with a good match between the observed and calculated intensity). For another five levels, the *J*-values have been revised compared to the original assignment given by Meggers et al. [9].

All new identifications were made with the help of the IDEN2 code [26]. Most of them involve two or more transitions with wavelengths satisfying the arithmetic relations between the observed and Ritz wave numbers within or close to the combined measurement uncertainties. They are also supported by the closeness of the experimental energy to that calculated here in the LSF, as well as in Ref. [3], where available. In most cases, the observed intensities are in good agreement with the calculated ones. In a few cases, this agreement is poor, which can be explained by large uncertainties in the calculated transition rates.

Five of the new line identifications listed in Table 1 are considered as tentative, as they are the sole lines defining the corresponding upper levels. One of them is the 6d^2^(^3^F)7s ${\;}^{4}{\text{F}}_{9/2}-6\text{d}{\;}^{2}\left({\;}^{3}\text{F}\right)7\text{p}{\;}^{4}{\text{G}}_{11/2}^{{}^{\circ}}$ transition, which was previously identified by Meggers et al. [9], with the line observed at 4682.16 Å. It is now identified with a stronger line observed at 4705.782(6) Å, which was previously classified as the $6\text{d}7\text{s}{\;}^{2}{\;}^{2}{\text{D}}_{5/2}-6{\text{d}}^{2}\left({\;}^{3}\text{F}\right)7\text{p}{\;}^{4}{\text{D}}_{7/2}^{{}^{\circ}}$ transition [9] with the upper level at 23,475.94 cm^−1^. As noted in Section 3.1, this level could not be observed in resonance excitation-ionization laser spectroscopy experiments [16] and thus was rejected here. The observed intensity of the 4705.782 Å line agrees much better with the present LSF calculation for the ${\;}^{4}{\text{F}}_{9/2}-{\;}^{4}{\text{G}}_{11/2}^{{}^{\circ}}$ transition than the 4682.16 Å line. The predicted intensity is defined by the calculated *A*-value; its accuracy is estimated to be very good (accuracy category B, corresponding to uncertainty ≤10%). However, the difference by a factor of two between the intensities of the previously and newly identified lines is within the uncertainty of the present intensity modeling (see Section 6), so the revised identification is considered as tentative. The line at 4682.16 Å is now unclassified. It is the strongest unidentified line in the list of observed Ac I lines in Ref. [9].

Another tentative identification deserving discussion involves the $6\text{d}7\text{s}\left({\;}^{3}\text{D}\right)7\text{p}{\;}^{4}{\text{D}}_{5/2}^{{}^{\circ}}$ level presently placed at 19,121.32 cm^−1^. It is based on a single moderately strong line observed at 5228.31 Å. Its observed intensity agrees very well with the presently predicted one, but the accuracy of the present *A*-value is very low (category C, uncertainty > 50%). The *A*-value calculated by Dzuba et al. [3] is five times smaller, but its accuracy is estimated to be similarly low. For this level, transition with the largest predicted *A*-value is predicted to be to the 6d^2^(^3^F)7s ^4^F_7/2_ level at 10,906.027(14) cm^−1^. Its wavelength, 12,169.09 Å (in air) is outside of the wavelength range covered by the study of Meggers et al. M57. Pending the observation of this transition, the present identification remains questionable.

Meggers et al. [9] had listed three observed lines classified as transitions from the odd-parity level near 30,396.6 cm^−1^ with *J* = 3/2. This level was labeled as $6\text{d}7\text{s}\left({\;}^{1}\text{D}\right)7\text{p }{\;}^{2}{\text{P}}_{3/2}^{{}^{\circ}}$. This level is now interpreted as $\;6{\text{d}}^{2}\left({\;}^{3}\text{F}\right)7\text{p}{\;}^{4}{\text{F}}_{3/2}^{{}^{\circ}}$ (with 76% of this term in its composition; see Table 2). This interpretation seemed questionable at first, because the strongest transition from this level was predicted to occur at 4720.274(6) Å, well within the range of Ref. [9], but was not listed among observed lines. It was found here that this line must have been masked by the strong Ac II line at 4720.16 Å.

Percentage compositions given in Table 2 include only the two leading components of the eigenvectors in *LS* coupling. In the even-parity system, the average purity of the eigenvectors (i.e., the arithmetic mean of the leading percentage) is 76% and 60% for the even and odd parity, respectively. In the even parity, even though a few levels have leading percentages less than 50%, all level labels corresponding to the leading percentage are unambiguous. However, in the odd parity, mixing between different eigenstates with the same *Jπ* symmetry (where *π* means parity) is much stronger. To provide unique labeling of all levels, in many cases it was necessary to use configuration and term designations of the second or third leading component of the eigenvector in the level labels given in the columns “Configuration” and “Term” of Table 2. These level labels have little physical meaning; they are used for bookkeeping only.

Table 2 includes all presently calculated levels up to the highest level tabulated in the work of Dzuba et al. [3], i.e., below 37 kK. As mentioned in Section 3.1, the two levels listed by Meggers et al. [9] at 23,475.94 cm^−1^ and 26,533.16 cm^−1^ have been rejected here. The first of them was already discussed above. As for the level at 26,533.16 cm^−1^, the lines assigned to this level in Ref. [9] imply that the only *J*-values possible for this level are 3/2 and 5/2. As can be seen in Table 2, there is no place for this level in the present interpretation of the level system. The closest unobserved odd-parity levels are predicted at 20,864 cm^−1^ and 33,617 cm^−1^ ($6\text{d}7\text{s}\left({\;}^{3}\text{D}\right)7\text{p}{\;}^{4}{\text{D}}_{3/2}^{{}^{\circ}}$and$6{\text{d}}^{2}\left({\;}^{3}\text{F}\right)7\text{p}{\;}^{4}{\text{D}}_{3/2}^{{}^{\circ}}$, respectively). This is much too far from the position suggested by Meggers et al. [9].

It must be noted that there are two factors greatly influencing the efficiency of IDEN2 in new line identifications: (1) accuracy of the computed transition probabilities used in the input, and (2) abundance of observed lines. With the generally low accuracy of *A*-values computed with Cowan’s codes and a small number of observed lines listed by Meggers et al. [9], it is very inefficient. A significant progress in the analysis of Ac I could be achieved if more observed lines were available. To estimate how many observed lines Meggers et al. [9] have omitted in their line list, I have scanned the top half of the portion of one photographic plate shown in Figure 2 of their paper. This endeavor was motivated by the figure caption stating that the very strong line at 4476 Å, marked on the figure, belongs to Ac I; no such line is listed in the tables of Meggers et al. [9]. Although the grainy and probably distorted photograph reproduced in the journal does not allow measuring the lines with high precision, the wavelengths could be determined with uncertainties of about 0.03 Å (0.003 nm). This was sufficient to identify numerous impurity lines of atomic iron (Fe I) and many lines that are probably due to Ac I. It turned out that the above-mentioned line at about 4476 Å, which is the strongest line in the figure, is the known strong line of atomic silver (Ag I) at 4476.040 Å [8]. This makes sense, as the figure displays a spectrum observed with an electric arc between silver electrodes with 0.5 mg of actinium implanted on the surface. This finding indicates that there are some errors in the paper of Meggers et al. [9]. Table 1 of Meggers et al. [9] contains 13 lines of Ac I within the region covered by their Figure 2, discussed here. A closer look reveals that, in this figure, the total number of lines having appearance similar to the known Ac I lines is 41. A few of them may be due to impurities, but most are probably due to Ac I. Thus, Meggers et al. [9] have listed only a quarter of all lines they observed. This suggests that the 150 photographic plates produced in the work of Meggers et al. [9] and stored in the NIST archives need to be reanalyzed.

## 4. Ac II

For Ra-like Ac II with the ground configuration [Rn]6d7s^2^, Meggers et al. [9] have listed a total of 296 observed lines, 221 of which were interpreted as transitions between the 65 energy levels found by those authors. In 1992, Blaise and Wyart [29] published a collection of atomic data for actinide spectra, in which they included the results of unpublished theoretical work of J.-F. Wyart on Ac II. He used a parametric fitting with Cowan’s computer codes [21] to interpret the energy structure. The [Rn](6d^2^ + 6d7s + 7s^2^ + 5f^2^ + 5f7p) and [Rn](5f6d + 5f7s + 6d7p + 7s7p) even- and odd-parity configuration groups were included in these calculations. In that work, Wyart rejected two levels, 5f7p ^3^G_5_ and $6\text{d}5\text{f}{\;}^{3}{\text{H}}_{5}^{{}^{\circ}}$, reported in Ref. [9]. While examining the several tens of unclassified Ac II lines observed by Meggers et al. [9], Wyart found four previously unknown levels, for which he could not find a theoretical interpretation. The principal ionization energy of Ac II was semiempirically determined by Martin et al. [30] to be 94,800(250) cm^−1^. For this determination, they extrapolated to Ac II the known differences of the quantum defects of the baricenters of the 7s^2^ and 7s8s configuration in the isoelectronic Ra I spectrum (Δn* = 1.053) and of the 7s and 8s configurations in the somewhat less-similar Ra II spectrum (Δn* = 1.063). The value of Δn* they adopted for Ac II was 1.055 ± 0.006, yielding the IE value quoted above.

The most precise theoretical calculations of the energy structure and transition properties of Ac II were made by Roberts et al. [31]. Unfortunately, that work includes only a few of the lowest energy levels and transitions between them. It does not help much in resolving the questions remaining after the work of Wyart described above. The earlier work of Quinet et al. [1] was made using Cowan’s suite of atomic codes [21] modified by inclusion of a model potential describing the effects of core polarization. In a semiempirical parametric fitting with these codes, those authors included the [Rn](6d^2^ + 6d7s + 7s^2^ + 7s8s) and [Rn](5f6d + 5f7s + 6d7p + 7s7p) even- and odd-parity configuration groups, i.e., the same sets of configurations as used by Wyart, except that instead of 5f^2^ and 5f7p, they included 7s8s in the even parity. They motivated the omission of the 5f7p configuration, which is partially known from the experiment [9], by its strong mixing with unknown configurations, such as 7p^2^, 6d8s, 6d7d, and 7s7d.

The small-scale multi-configuration Dirac–Fock calculations of Ürer and Özdemir [32] included only the 56 levels of the same eight configurations as considered by Quinet et al. [1]. Since these calculations were ab initio, i.e., they did not include any semiempirical adjustments or core-polarization corrections, they are very inaccurate and inferior to the calculations of Ref. [1].

To make some progress in the analysis, new parametric calculations were made in the present work with another version of Cowan’s codes [22]. The following configuration sets were included: [Rn](6d^2^ + 6d7d + 6d8d + 6d9d + 6d5g + 7s^2^ + 7s8s + 7s9s + 7s7d + 7s8d + 7s9d + 7s5g + 6d7s + 6d8s + 6d9s + 5f^2^ + 5f7p + 5f8p + 5f9p + 7p^2^) and [Rn](7s7p + 7s8p + 7s9p + 6d7p + 6d8p + 6d9p + 5f7s + 5f8s + 5f9s + 5f6d + 5f7d + 5f8d + 5f9d + 7s6h + 6d6h + 5f5g) in the even- and odd-parity sets, respectively. The previous LSF calculations for neutral Ac made with the help of the data from the large-scale ab initio calculations of Dzuba et al. [3] provided vital clues about the locations of the experimentally unknown configurations. Their average energies have been adjusted from the ab initio HFR values by the same amounts as configurations involving similar subshells in Ac I. As in Ac I, similar Slater parameters in all configurations were linked in groups, so that fitting of the structure of experimentally known lowest excited configurations automatically improved predictions of internal structure of the unknown highly excited configurations. The LSF calculations were conducted in the same iterative manner as in Ac I, by transferring the fitted parameters to the RCG code, calculating the transition probabilities with these fitted parameters, loading them into the input files of the IDE2 code, and searching for new levels having predicted transition wavelengths and intensities agreeing with observed lines. If one or more new levels were found, they were introduced in the LSF, and the entire procedure was repeated.

In this way, it was possible to identify 16 new energy levels describing 33 observed, previously unclassified lines. Four of these new levels are tentative, as they are based on one observed line each. One new level (at 64,154.91 cm^−1^), based on two observed lines, is also treated as questionable, because the strongest transition predicted to occur from it at 3249.366(9) Å (down to the level at 33,388.554 cm^−1^) is not present in the tables of Meggers et al. [9]. Perhaps, it was mistaken for a La II line at 3249.35 Å [8], as lanthanum is listed in Ref. [9] as one of the many impurities. In addition, the original identification of the 5f7p ^3^G_5_ level [9], which was rejected by Wyart (see above), was found to be correct and has been reinstated. For 11 levels, the previous level designation (configuration, term, or *J-*value) from Blaise and Wyart [29] has been revised. One level listed by Meggers et al. [9] at 60,063.0 cm^−1^ and designated as *e* ^3^D_1_ has been discarded, and the two lines attributed to it in Ref. [9] have been reclassified as transitions from other levels.

In the final LSF calculation, 45 experimentally known levels of even parity were fitted with an rms of the differences (observed minus calculated energies) of 128 cm^−1^. For the 38 known levels of odd parity, this rms difference is 281 cm^−1^. For the levels common with those tabulated by Roberts et al. [31] (13 even and 3 odd), the rms difference of the present LSF calculation from the experiment is 76 cm^−1^, to be compared with the corresponding value from Roberts et al., 456 cm^−1^. Compared to these numbers, the results of the LSF of Quinet et al. [1] are much worse: 1162 cm^−1^ for 18 even levels and 426 cm^−1^ for 37 odd levels. Note that, according to the present analysis, the two lowest experimental odd levels with *J* = 2 were interchanged in the calculations of Quinet et al. [1], as well as in the tabulated results of Roberts et al. [31], since their designations were interchanged in the works of Meggers et al. [9] and Blaise and Wyart [29]. In addition, note that, in the LSF of Quinet et al. [1], the experimental odd-parity level at 36,144.35 cm^−1^ [9] (*J* = 3) was mistaken as 35,144.35 cm^−1^. From the above, it is evident that the present parametric calculation is superior to all previous calculations in the accuracy of predicted energy levels.

Transition probabilities have been calculated with Cowan’s codes [22] by using the fitted Slater parameters from the LSF. In this calculation, the values of the s–p and p–d E1 transition matrix elements were scaled by a factor of 0.9284 to bring the calculated *A*-values in agreement with the calculation of Roberts et al. [31] for the strongest transitions. This scaling factor is comparable to the one used in the Ac I calculation (0.811; see Section 3.3). As in the Ac I calculation, the d–f and f–g E1 transition matrix elements were scaled by a factor of 0.8233 taken from the analysis of Ac III data (see Section 5). Since the calculations of Quinet et al. [1] and of Ürer and Özdemir [32] were found to be too inaccurate, the only available benchmark for comparison with the present calculation is the work of Roberts et al. [31]. Out of the total of 11 *A*-values tabulated by them, those of the four strongest transitions with the presently calculated line strengths *S* > 2 a.u. agree with the present ones within 11% on average. For the five weaker transitions with *S* between 0.1 a.u. and 2 a.u., the average ratio to the present values is a factor of two, and for the two weakest transitions with *S* ≈ 0.04 a.u., the average ratio is a factor of 9. The uncertainties of all presently calculated *A*-values were roughly estimated by extrapolating this trend to all transitions considered in the present work.

All 296 observed lines attributed to Ac II by Meggers et al. [9] are listed in Table 4. For 270 of these lines, the table includes the lower and upper level classifications and Ritz wavelengths (one of these lines is doubly classified). For 245 of these classified lines, the table also includes a critically evaluated *A*-value with its uncertainty expressed in terms of the NIST accuracy category. Most of these *A*-values are from the present calculations, only four being from Roberts et al. [31]. In addition to E1 transitions, all potentially important M1 and E2 transition probabilities have been calculated in this work. To the author’s knowledge, no data for these transitions have been previously reported. For these transitions, as in the Ac I calculations described in Section 3.3, the method of Monte Carlo random trials [27] was used for estimation of uncertainties of the calculated *A*-values. As in Ac I, 100 random trials were used, in which the Slater parameters were randomly varied around their values from the LSF, and the variance of the E2 transition matrix elements was assumed to be 15%. In Ac II, unlike Ac I, there are two metastable or anomalously long-lived odd-parity levels with large *J*-values. However, these levels have not been found experimentally, so it was not possible to include the forbidden transitions from these levels in Table 4. Thus, all predicted forbidden transitions included in this table are between even-parity levels. These transitions have branching fractions greater than 2%. A transition is deemed to be of a mixed type (M1 + E2) if the contribution of one of the types to the total *A*-value exceeds 2%.

The experimental and calculated energy levels of Ac II are listed in Table 5. There are now 83 experimentally known Ac II levels (45 even and 38 odd). The uncertainties given for the level values in Table 5 pertain to the separations of the levels from the 6d^2 3^F_2_ level at 13,236.418 cm^−1^. This level was chosen as the base for the determination of uncertainties, since it participates in the largest number of observed lines (19). The uncertainty of the excitation energy of any level from the ground level can be determined as a combination in quadrature of the uncertainty of this level given in Table 5 and the uncertainty of the ground level, 0.03 cm^−1^.

Table 5 also includes all levels predicted below the highest experimentally known levels in each parity (68,692.14 cm^−1^ and 56,582.72 cm^−1^ for the even and odd parity, respectively). The data from the present LSF calculations are also included in the table: energies, percentage compositions (up to three leading terms with percentages greater than 5%), Landé *g*_*J*_-factors, and radiative lifetimes. The latter were calculated by summing up all presently considered radiative decay branches, including E1, M1, and E2 transitions. According to the present calculation, the lowest excited state 6d7s ^3^D_1_ at 4739.631(33) cm^−1^ is extremely long-lived. Its radiative lifetime, determined by the M1 transition to the ground state at 21,098.69(15) Å, is about 3 × 10^6^ years (with an estimated uncertainty of 50%). This value does not account for hyperfine-induced transitions that must substantially reduce it in odd isotopes of actinium. The longest-lived isotope of actinium is ^227^Ac with a half-life of 22 years, which sets a practical limitation on the lifetime of any excited state. The lifetime of the 6d^2 3^P_2_ level at 19,202.962(33) cm^−1^, which is of interest for studies of parity non-conservation [31], is presently calculated to be 0.215(10) s. This value agrees with the result of Roberts et al. [31], which is about 0.2 s.

The presently calculated Landé factors included in Table 5 agree with those previously calculated by Quinet et al. [1], with rms differences of 0.017 in the even parity and 0.06 in the odd parity. In the absence of a better benchmark for comparison, these rms differences can be adopted as the uncertainties of the present values.

No attempt was made here to re-evaluate the IE of Ac II. Thus, the recommended value of IE included in Table 5 is the semiempirical one quoted from Martin et al. [30].

The final fitted values of the Slater parameters resulting from the present LSF for Ac II are listed in Table 6.

## 5. Ac III

The ground state of francium-like Ac III is [Rn]7s. Meggers et al. [9] have identified eight lines of Ac III, from which they determined the values of six excited levels. All these identifications have been confirmed here. The wavelengths reported by Meggers et al. [9] are internally consistent: they deviate from the Ritz values by less than 0.003 Å. However, the strong polar effect in the setup of Meggers et al. [9] may have led to a sizeable systematic shift in the measured wavelengths. Thus, the uncertainties of these measurements are conservatively estimated to be 0.013 Å for the lines above 3000 Å. For lines with shorter wavelengths, which are likely to have been measured in both the first and second orders of diffraction, a smaller uncertainty of 0.006 Å is assumed here. The list of observed lines of Ac III is given in Table 7.

As for Ac I and Ac II, the experimental energy levels have been redetermined here from the eight observed spectral lines by means of a least-squares level optimization with the code LOPT [28]. The list of the newly optimized energy levels of Ac III is given in Table 6. Separations of the optimized excited levels from the 6d ^2^D_5/2_ level at 4203.89 cm^−1^ have uncertainties in the range from 0.04 cm^−1^ to 0.10 cm^−1^. These uncertainties are given in Table 6. To obtain the uncertainties of excitation energies from the ground level (7s ^2^S_1/2_), they must be combined in quadrature with the uncertainty of the ground level, 0.09 cm^−1^.

On the theoretical side, the most precise reported calculations of energy levels and E1 transition rates are those of Roberts et al. [33], of Safronova et al. [34], and of Migdalek and Glowacz-Proszkiewicz [35]. For Ac III, E1 transition rates of Roberts et al. [33] and Safronova et al. [34] agree with each other within 3% on average. For only two longest-wavelength transitions $\left(6\text{d}{\;}^{2}{\text{D}}_{5/2}-5\text{f}{\;}^{2}{\text{F}}_{5/2,7/2}^{{}^{\circ}}\right)$, the difference between these two calculations reaches 5%. The *A* values of Roberts et al. [33] have been adopted here as recommended values. Their uncertainties are assigned according to the comparison outlined above. The *A* values of Migdalek and Glowacz-Proszkiewicz [35] deviate from those of Roberts et al. [33] by 5% on average. For the *A* values of Biémont et al. [36], the average deviations from Ref. [33] are slightly larger; 9% on average. For comparison, the *A* values computed by Ürer and Özdemir [37] are systematically lower than the reference values by (53 ± 30)% on average. These primitive Dirac–Fock calculations included only six configurations of even parity and five configurations of odd parity. The poor quality of the results speaks for itself.

Although Cowan-code calculations cannot compete in accuracy with the large-scale calculations of Roberts et al. [33] and Safronova et al. [34], such calculations were made in this work with the sole purpose of evaluation of systematic errors in the transition matrix elements computed with Cowan’s codes. The scaling factors needed to bring the calculated E1 *A* values were 0.9510 for the s–p and p–d transitions and 0.8233 for the d–f and f–g transitions. The latter factor was used in the Ac I and Ac II calculations, where no reference values are available for an independent estimation.

Parity-forbidden E2 and M1 transition rates for transitions from the lowest two excited levels of Ac III have been reported by Safronova et al. [38]. These authors have included their estimated uncertainties for the *A* values and radiative lifetimes. These uncertainties are between 0.4% and 1.5%. The reference values taken from Ref. [38] have been used here to evaluate the systematic errors in the E2 transition matrix elements computed with Cowan’s codes. It turned out that, unlike the E1 transitions, the E2 *A* values computed with Cowan’s code agree with the reference values within a few percent with no discernible systematic difference. This observation in Ac III was extrapolated to the other Ac spectra, so that no scaling was applied to the E2 transition matrix elements in any of the spectra studied in this work.

The Landé *g*_*J*_ factors of the three lowest levels of Ac III were precisely calculated by Gossel et al. [39]. The rms difference of the *g*_*J*_ values calculated in that work from much more precise experimental values for Rb, Cs, Ba^+^, and Fr is 3 × 10^−5^, which can be adopted as an estimate of uncertainty for the Ac III values.

The currently recommended values of the principal ionization energy (IE) of Ac III, 140,590 cm^−1^ [8], is quoted from Migdalek and Glowacz-Proszkiewicz [35]. Its estimated uncertainty, 160 cm^−1^, was derived from isoelectronic comparisons made in my unpublished research on the Fr isoelectronic sequence made in 2011. The newer calculations of Roberts et al. [33], as well as the calculations of Safronova et al. [34], which were overlooked in my early research, make it possible to establish a more precise value of the IE. A fairly extensive study of these data was undertaken in the present work. Unfortunately, the data of Migdalek and Glowacz-Proszkiewicz [35], as well as those of Safronova et al. [34], were found to contain errors that make them not smooth along the isoelectronic sequence.

The coefficient *b* given below Equation (5) of Migdalek and Glowacz-Proszkiewicz [35] has a misprint in the power of 10: it must be −1, not −2. However, even with the corrected *b* value, the values of the dipole polarizability *α* in Table 1 of that paper cannot be reproduced with the given equation. There is a discontinuity in the *α* values between Ac and Th, which is revealed in the residual differences between the *α* values of Table 1 of Ref. [35] and those computed with Equation (5) of that paper. The cause of this discontinuity may be in the values of the mean radii given in the same table, which are supposed to be fitted for Fr I through Th IV and extrapolated to the higher ions.

In the paper of Safronova et al. [34], there are several inconsistencies between their Table I and Table II. For example, for Fr I, the excitation energies of 6d_3/2_ and 6d_5/2_ given in Table II disagree with Table I by 286 cm^−1^ and 208 cm^−1^, respectively. For the U VI 7p_1/2_ and 7p_3/2_ levels, the disagreement is much larger: 519 cm^−1^ and 1450 cm^−1^, respectively. Table I of Ref. [34] lists the values of several computed quantities representing various contributions to the total binding energy. Some of these contributions are relatively small and are expected to vary smoothly along the isoelectronic sequence. However, this smoothness is disrupted by the abnormally large values of the parameters *E*^(3)^ and $E_{\text{extra }}^{\left(3\right)}$ for the 7p_3/2_ level of Pa V. An isoelectronic comparison of the binding energies of the 6d_3/2_ and 6d_5/2_ levels computed with two methods by Safronova et al. [34] with those of Roberts et al. [33] reveals that the latter are likely to be too high by about 1000 cm^−1^ in Pa V.

Despite the problems discussed above, it was possible to interpolate the differences between the experimental and theoretical values of quantum defects for the 7s_1/2_, 6d_3/2,5/2_, 7p_1/2,3/2_, and 5f_5/2,7/2_ levels along the Fr isoelectronic sequence from Fr I to U VI and derive improved values of the IE for Ac III, Th IV, Pa V, and U VI. These values are 140,630(50) cm^−1^, 230,973(14)) cm^−1^, 361,690(200) cm^−1^, and 506,400(50) cm^−1^, equivalent to 17.436(6) eV, 28.6371(17) eV, 44.844(25) eV, and 62.786(6) eV, respectively. A detailed description of these isoelectronic interpolations will be the subject of a future paper. The above IE value for Th IV has been derived from a newly reoptimized set of experimental energy levels based on the wavelengths reported by Klinkenberg [40]. The series of the *n*s_1/2_ (*n* = 7–10) energy levels was used in this determination, which employed a fitting of the extended Ritz quantum-defect expansion formula (see Kramida [12]) and comparisons with similar series in isoelectronic Fr I and Ra I.

A more precise determination of the IE could be made in the future, when more accurate calculations become available. Such calculations are desirable for the entire sequence from Fr I up to Np VII (for the latter spectrum, the only data available at present are those of Roberts et al. [33]). These calculations should be smooth along the isoelectronic sequence and include not only the levels listed above, but also 7d (*J* = 3/2, 5/2), 8p (*J* = 1/2, 3/2), and 8s (*J* = 1/2). These levels are precisely known experimentally for Fr I and Ra II, but in U VI their experimental values are provided with a rather low precision by the beam-foil study of Church et al. [41]. The abnormally large deviations of quantum defects of the 8p levels from the calculations of Roberts et al. [33] make the identifications of Church et al. [41] questionable. In terms of excitation energy, the discrepancy is about 4600(1600) cm^−1^ for the 8p_1/2_ level. More precise calculations could confirm or disprove this experimental identification.

The lists of observed spectral lines and energy levels of Ac III are given in Tables 7 and 8, respectively. Data for predicted forbidden transitions of Ac III are included in Table 7 for completeness. The lifetime values included in Table 8 are computed as sums of the E1, M1, and E2 radiative decay channels. The lifetime value for the 6d ^2^D_5/2_ level, 2.305(34) s, differs slightly from the value originally reported by Safronova et al. [38], 2.326(34) s, possibly because in the present work the *A* values have been adjusted to experimental transition energies. The original values of the reduced transition matrix elements reported in Ref. [38] have been used here.

## 6. Reduction of Observed Line Intensities

Meggers et al. [9] have reported five sets of observed line intensities from the five types of light sources they used: an arc and a spark between silver electrodes, an arc and a spark between copper electrodes, and a hollow-cathode discharge. These light sources are denoted hereafter as “Ag arc”, “Ag spark”, “Cu arc”, “Cu spark”, and ”HC”, respectively. Small amounts of actinium were introduced into these light sources by soaking porous tips of the electrodes in a nitrate solution of Ac or by precipitating a similar solution on the bottom of the hollow cathode. No information was given by Meggers et al. [9] about the methods used in reduction of the observed intensities. They mentioned that several different types of photographic plates were used in different recordings: Eastman Kodak 103-F, 103-C, 103a-C, 103a-F, 103a-F (UV), I-N, and I-Q. For the most informative recordings, the 103a-F (UV) plates were used for the ultraviolet region, 103a-F for near ultraviolet and visible, I-N for red and adjacent infrared, and I-Q for longer wavelengths. It was noted that, in some exposures, “overlapping spectral orders were differentiated by supporting appropriate gelatine filters in front of the photographic plates to absorb portions of the slit images”. The intensity values were given in the tables of Meggers et al. [9] on an apparently linear scale (in terms of exposure) with values between 1 and 5000. However, no information about the dependence of the overall sensitivity of the multiple setups on wavelength is available. The different light sources had notably different temperatures, which was manifested in enhanced intensities of Ac II and Ac III lines in sparks. Thus, reduction of all these intensity measurements to a common scale is a nontrivial task.

To achieve that, the present work uses the method suggested by Kramida [12] and described in more detail in later publications (see, e.g., Kramida et al. [42,43]). This method is based on the assumption of the Boltzmann distribution for the populations of excited levels and neglects self-absorption. The important prerequisite for this method to work is the availability of reliable *A* values for most of the lines throughout the entire spectral range of the observations. These requirements are likely to have have been met: extensive sets of fairly accurate *A* values are available for all three spectra (Ac I, Ac II, and Ac III; see Tables 1, 5 and 7), the tiny amounts of Ac introduced into the discharges make self-absorption to be unlikely, and the level populations in all types of the light sources used by Meggers et al. [9] are sufficiently close to local thermodynamic equilibrium.

The effective excitation temperatures determined from the slopes of Boltzmann plots (see [12,42,43]) in the various light sources used by Meggers et al. [9] are listed in Table 9 for each Ac spectrum. From the scatter of data points in the plots, uncertainties of these values are can be roughly estimated as about 20%.

As can be seen from Table 9, the observed spectra of different ions exhibit different excitation temperatures in the same light source. This is due to the different spatial origin of the spectra, which can be seen in Figures 2 and 3 of Meggers et al. [9]: lines of Ac I, Ac II, and Ac III have distinctly different distributions of intensities along the line height. For Ac III spectra taken with the Cu arc and HC discharges, it was not possible to determine the temperature, because only a few transitions with relatively close upper-level energies were observed in these spectra. For these spectra, the slope of the Boltzmann plots was fixed at zero in the intensity-reduction procedure.

**Figure 6.**
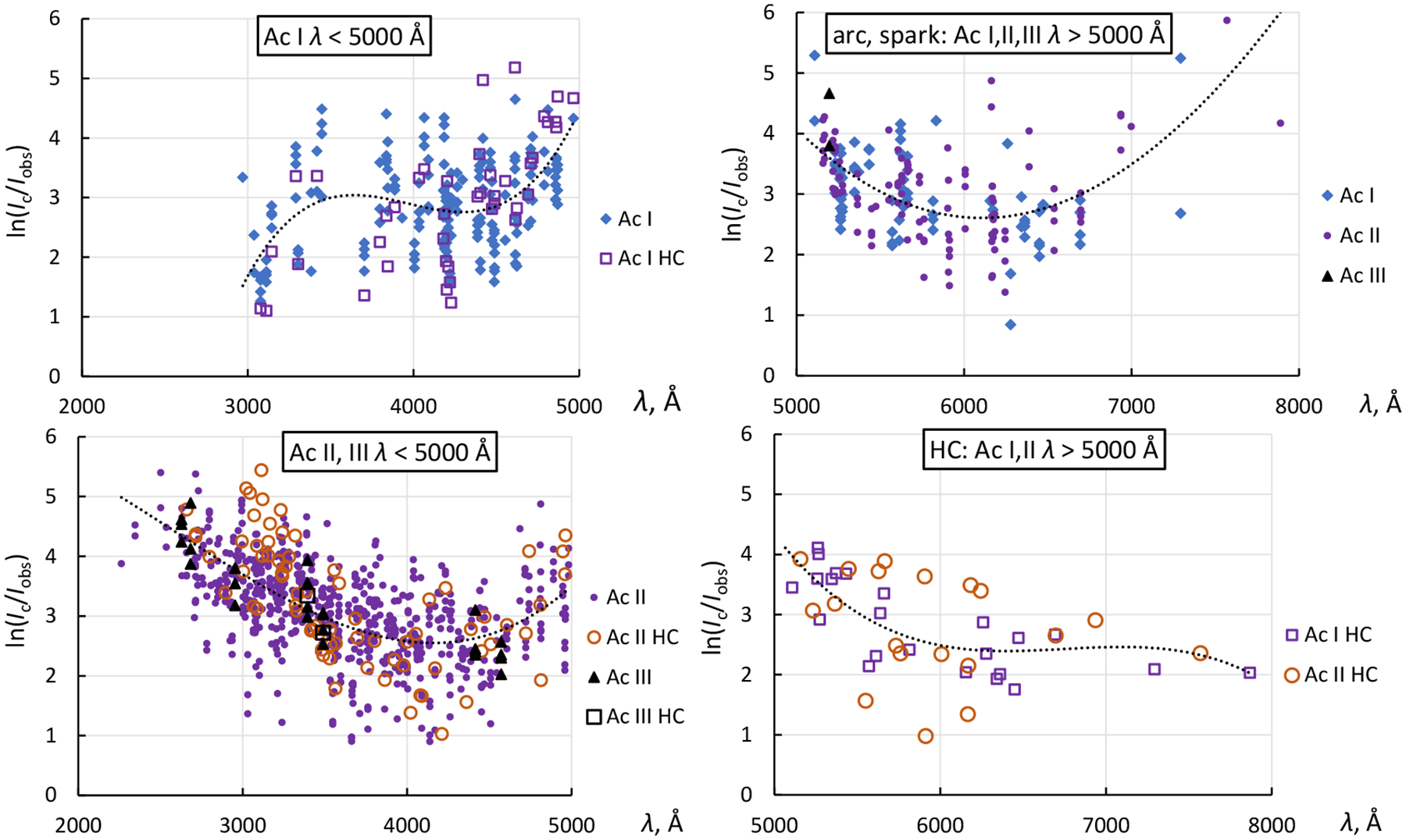
Inverse logarithmic spectral response functions for observations of Meggers et al. [9]. Top left: Observed Ac I short-wavelength line intensities in the Ag and Cu arc and spark spectra (labeled “Ac I”) and in the HC spectrum (labeled “Ac I HC”). Bottom left: Observed Ac II and Ac III short-wavelength line intensities in the Ag and Cu arc and spark spectra (labeled “Ac II” and “Ac III”) and in the HC spectrum (labeled “Ac II HC” and “Ac III HC”). Top right: Observed Ac I, Ac II, and Ac III long-wavelength line intensities in the Ag and Cu arc and spark spectra (labeled “Ac I”, “Ac II”, and ”Ac III”). Bottom right: Observed Ac I and Ac II long-wavelength line intensities in the HC spectrum.

The logarithmic inverse spectral response plots (see [12,42,43]) derived from the observed intensities are displayed in Figure 6. The inverse spectral response function *R*(*λ*) is defined as *R*(*λ*) = ln(*I*_c_/*I*_obs_), where *λ* is the observed wavelength, *I*_c_ is the calculated intensity, and *I*_obs_ is the observed intensity. To remove the wavelength-dependence of the spectral response of the instrument from the observed intensities, the latter are multiplied by exp(*R*(*λ*)).

It was found that Meggers et al. [9] used different intensity-reduction procedures in the short-wavelength (*λ* < 5000 Å) and long-wavelength (*λ* > 5000 Å) regions. However, in each of these regions, the same reduction procedure was applied to certain groups of spectra. This can be seen, for example, in the top-left panel of Figure 6, showing the behavior of the observed Ac I intensities. There is no discernible difference in the shape of *R*(*λ*) between the Ag arc, Ag spark, Cu arc, and Cu spark spectra, so they are all displayed with the same symbol (full rhombus). The ratios *I*_c_/*I*_obs_ for the HC intensities may be perceived from the plot as slightly deviating from the overall fit shown by the dotted line, but these deviations are within the range of scatter of the data points. Thus, a common *R*(*λ*) function shown by the dotted curve was used to correct the observed intensities in all these spectra.

A similar comparison is shown in the same wavelength range for the Ac II and Ac III spectra in the bottom-left panel of Figure 6. Again, the general behavior of the *I*_c_/*I*_obs_ values is very similar for both Ac II and Ac III spectra recorded with Ag and Cu arcs and sparks and with HC. However, this behavior is very different from the one observed for the Ac I spectrum: at the shortest wavelengths below 3500 Å, the observed Ac II and Ac III intensities appear to be strongly suppressed, so that larger *R*(*λ*) values are needed to bring them in agreement with the calculated intensities. This suppression may have been caused by the use of filters to suppress higher orders of diffraction. Again, a common *R*(*λ*) function shown by the dotted curve was used to correct the observed intensities in all spectra included in this panel.

For the long-wavelength region above 5000 Å, a different division is observed between the various spectra. As shown in the top-right panel of Figure 6, all three actinium spectra appear to have the same scale of intensities observed in the arc and spark recordings. The overall shape of the *R*(*λ*) function shown by the dotted curve, with a minimum near 6000 Å, is very reasonable, since the grating used in these recordings was blazed at this wavelength. However, the HC recordings (bottom-right panel of Figure 6) display a very different behavior of *R*(*λ*) at the longest wavelengths above 6500 Å. Near 8000 AA, the observed HC relative intensities are much greater (by about two orders of magnitude) compared to the arc and spark intensities. The exact cause of the increased HC intensities at longer wavelengths is unknown. It might have been caused by the use of a different type of photographic plates in these recordings.

When the shapes of the *R*(*λ*) function are established for each observed spectrum, and the effective temperatures are determined from Boltzmann plots, reduction of the observed intensities to a common scale corresponding to a chosen light source is straight-forward (see a detailed description of this procedure in Kramida et al. [43]). For Ac I, the observed intensities given in Table 1 have been reduced to the same scale as established for the Ag arc recordings with an effective temperature of 0.51 eV. For Ac II and Ac III intensities given in Tables 4 and 7, respectively, the scale was based on the Ag spark observations with effective temperatures of 0.77 eV and 2.18 eV for Ac II and Ac III, respectively (see Table 9). Most of the tabulated intensity values are averages of several reduced intensity values from up to five observations in different light sources. These intensity values are expected to be accurate within a factor of two or three, on average. In principle, they allow the *gA* values to be derived from them (with that low accuracy) by constructing a Boltzmann plot with the temperatures given above (see Equation (1)). The scatter of the data points in Figure 6 suggests that a few of the intensity values may be in error by a factor of 10, or even more.

## 7. Conclusions

As a result of this work, several tens of new identifications have been made in the previously published Ac I and Ac II line lists of Meggers et al. [9] (16 in Ac I and 36 in Ac II). In Ac I, 16 new energy levels have been found, and the *J* values of 5 previously reported levels have been revised. In Ac II, 16 new energy levels have been established; one level listed by Meggers et al. [9] but discarded by Blaise and Wyart [29] has been reinstated, and one of the levels listed by Meggers et al. [9] has been discarded. New parametric least-squares fitting calculations with Cowan’s codes [21,22] have been made for both Ac I and Ac II, providing eigenvector percentage compositions that involve revised classifications for several levels. These calculations have also provided a large number of new *gA* values in both Ac I and Ac II. The principal ionization energies (IE) of Ac I and Ac III have been redetermined with improved precision. As a byproduct, improved IE values have been determined for three other Fr-like actinide ions: Th IV, Pa V, and U VI.

The tables provided in this work represent the currently recommended reference data on energy levels, spectral lines, and transition probabilities of Ac I–III intended for inclusion in a future release of the NIST ASD [8].

Further progress in the knowledge of Ac spectra is impeded by scarcity of available experimental data. A partial analysis of a small part of photographic recordings of Meggers et al. [9] stored in the NIST archives indicates that only about a quarter of all Ac lines present on these plates are included in the published line lists. A reanalysis of these plates may be warranted. New laser-spectroscopy studies could test the validity of some tentative identifications in Ac I. Zeeman-effect patterns have never been experimentally studied in any Ac spectrum. Such studies could provide information on Landé factors, which are crucial in interpretation of energy levels. On the theoretical side, improved and extended large-scale calculations of all three first spectra of Ac could be useful in elucidating the intricate level structure of Ac I and Ac II riddled with strong configuration interactions and in precise determination of ionization energies of Ac III and other Fr-like ions.

## Supplementary Material

machine-readable_tables

## Figures and Tables

**Table 1.** Spectral lines of Ac I.

λ_obs_^a^	λ_Ritz_^a^	Δλ_O-R_^b^	*σ*_obs_^c^	*I*_obs_^d^	Lower Level		Upper Level		*E*_low_^e^	*E*_upp_^e^	*A* ^f^	Acc.^g^	Type^h^	TP	Line	Notes^j^
(Å)	(Å)	(Å)	(cm^−1^)	(arb.u.)	Configuration	Term_*J*_	Configuration	Term_*J*_	(cm^−1^)	(cm^−1^)	(s^−1^)			Ref.^i^	Ref.^i^	
2968.819(4)	2968.8142(15)	0.005	33,673.59	23	6d7s^2^	^2^D_3/2_	6d^2^(^3^P)7p	${\;}^{4}{\text{D}}_{3/2}^{{}^{\circ}}$	0.000	33,673.650	9.e+06	E		TW	M	
3036.930(4)	3036.9294(16)	0.001	32,918.40	87	6d7s^2^	^2^D_3/2_	6d^2^(^3^P)7p	${\;}^{2}{\text{D}}_{3/2}^{{}^{\circ}}$	0.000	32,918.416	6.e+06	E		TW	M	
3076.440(4)	3076.4385(18)	0.002	32,495.66	280	6d7s^2^	^2^D_3/2_	6d^2^(^3^F)7p	${\;}^{2}{\text{F}}_{5/2}^{{}^{\circ}}$	0.000	32,495.679	5.e+06	E		TW	M	
3082.957(6)	3082.9507(16)	0.006	32,426.97	16	6d7s^2^	^2^D_5/2_	6d^2^(^3^F)7p	${\;}^{4}{\text{D}}_{5/2}^{{}^{\circ}}$	2231.432	34,658.472					M	
3109.330(13)			32,151.94	83											M	
3111.570(4)	3111.5683(17)	0.001	32,128.80	420	6d7s^2^	^2^D_5/2_	6d^2^(^1^G)7p	${\;}^{2}{\text{G}}_{7/2}^{{}^{\circ}}$	2231.432	34,360.247	1.0e+07	E		TW	M	
3140.720(13)			31,830.61	100											M	
3143.710(4)	3143.7088(18)	0.001	31,800.34	1100	6d7s^2^	^2^D_3/2_	5f7s^2^	${\;}^{2}{\text{F}}_{5/2}^{{}^{\circ}}$	0.000	31,800.350	4.3e+07	D+		TW	M	
3171.170(4)	3171.1658(18)	0.004	31,524.98	1200	6d7s^2^	^2^D_5/2_	6d^2^(^3^F)7p	${\;}^{2}{\text{F}}_{7/2}^{{}^{\circ}}$	2231.432	33,756.455					M	
3174.224(4)	3174.2212(16)	0.003	31,494.65	160	6d7s^2^	^2^D_3/2_	6d^2^(^3^F)7p	${\;}^{4}{\text{F}}_{5/2}^{{}^{\circ}}$	0.000	31,494.679					M	
…																
6340.100(13)	6340.104(11)	−0.004	15,768.26	200cs	6d7s(^3^D)7p	${\;}^{4}{\text{F}}_{7/2}^{{}^{\circ}}$	6d7s(^3^D)8s	^4^D_5/2_	17,683.869	33,452.12	1.52e+07	B		TW	M	N
…																
13,370.05(7)	13,370.05(7)		7477.36	1500	6d7s^2^	^2^D_3/2_	7s^2^7p	${\;}^{2}{\text{P}}_{1/2}^{{}^{\circ}}$	0.000	7477.36	1.497e+06	A+		Z	Z	
	44,814.27(16)				6d7s^2^	^2^D_3/2_	6d7s^2^	^2^D_5/2_	0.000	2231.432	1.204e−01	AA	M1	TW		
	85,321.3(11)				6d^2^(^3^F)7s	^4^F_7/2_	6d^2^(^3^F)7s	^4^F_9/2_	10,906.027	12,078.067	4.11e−02	A+	M1	TW		
	95,929.0(11)				6d^2^(^3^F)7s	^4^F_5/2_	6d^2^(^3^F)7s	^4^F_7/2_	9863.589	10,906.027	4.53e−02	A+	M1	TW		
	154,727(3)				6d^2^(^3^F)7s	^4^F_3/2_	6d^2^(^3^F)7s	^4^F_5/2_	9217.288	9863.589	1.071e−02	A+	M1	TW		

a Observed and Ritz wavelengths are given in standard air. Conversion between air and vacuum wavelengths was made with the five-parameter formula for the dispersion of air from Peck and Reeder [11].

b The difference between the observed and Ritz wavelengths. Blank for lines with unmeasured wavelength and for the lines that solely determine one of the energy levels of the transition.

c Transition wave number in vacuum.

d Observed intensity on an arbitrary scale, which is linear in terms of the energy flux under the line contour (see Section 6). The symbols after the numbers denote the character of the line: c—complex structure; D—double line; l—shaded on the long-wavelength side; m—masked by a stronger line; s—shaded on the short-wavelength side; :—the wavelength was not measured (the given value is a rounded Ritz wavelength).

e The optimized energies of the lower and upper levels of the transition. These values correspond to those given in Table 2.

f Transition probability. Exponential notation is used (e.g., “9.e+06” means “9. × 10^6^”).

g Accuracy of the transition probability. The code symbols for the accuracy are defined in the NIST ASD [8] (see https://physics.nist.gov/PhysRefData/ASD/Html/lineshelp.html#OUTACC, accessed on 25 March 2022).

h Transition type: blank—electric dipole; M1—magnetic dipole; E2—electric quadrupole; M1 + E2—mixed type (both types contribute more than 2% to the total *A* value given here).

i Code for references: D—Dzuba et al. [3]; Dn—values of Ref. [3] renormalized using the radiative lifetimes of Ref. [2]; M—Meggers et al. [9], Z—Zhang et al. [2]; TW—this work.

j Notes: D—the previous classification (of Ref. [9]) has been discarded; M—the line was masked by a much stronger Ac II line on the spectrograms of Ref. [9]; N—a newly classified line; R—the previous classification (of Ref. [9]) has been revised; T—a new tentative identification.

(Only a small portion of this table is given here for guidance to its content. The full version is available in machine-readable format in Table S1 of the Supplementary Online Materials, file Table S1.txt. The format of the supplementary table is slightly different: the uncertainties of *λ*_obs_ and *λ*_Ritz_ are given in separate columns instead of parentheses; the *J*-values of the lower and upper levels are given in separate columns; all notation is given inline with no superscripts or subscripts; the odd-parity symbol is replaced with the asterisk).

**Table 2.** Energy levels of Ac I.

*E*_exp_	Unc.	Configuration	Term	*J*	Leading Percentages^a^				*E*_calc_^b^ (cm^−1^)		*g*_calc_^c^		τ^d^(ns)			Ref.^e^	Notes^f^
(cm^−1^)	(cm^−1^)								TW	[3]	TW	[3]	TW	u%	other^d^		
0.000	0.000	6d7s^2^	^2^D	3/2	94				87	0	0.799	0.8001				M	
2231.432	0.008	6d7s^2^	^2^D	5/2	93				2157	2339	1.200	1.2002	8.30e+09	1.0		TW,M	
7477.36	0.04	7s^2^7p	^2^P°	1/2	90	6	6d7s(^3^D)7p	^2^P°	7422	7565	0.664	0.6626	690	10	668(11)^Z^	Z	
9217.288	0.013	6d^2^(^3^F)7s	^4^F	3/2	93	5	6d^2^(^1^D)7s	^2^D	8766	8989	0.425	0.4088	1.0e+07	50		TW,M	
9863.589	0.012	6d^2^(^3^F)7s	^4^F	5/2	91				9659	9288	1.036	1.0298	9.7e+09	38		TW,M	
10,906.027	0.014	6d^2^(^3^F)7s	^4^F	7/2	98				11,111	9974	1.235	1.2333	1.27e+10	11		TW,M	
12,078.067	0.019	6d^2^(^3^F)7s	^4^F	9/2	93	6	6d^2^(^1^G)7s	^2^G	12,564	11,726	1.320	1.3143	1.5e+10	24		TW,M	
12,276.591	0.020	7s^2^7p	^2^P°	3/2	84	10	6d7s(^3^D)7p	^2^P°	12,443	12,345	1.334	1.3332	280	10	255(7)^Z^	Z	
		6d^2^(^3^P)7s	^4^P	1/2	91	6	6d^2^(^1^S)7s	^2^S	12,404	12,583	2.601	2.6295	2.7e+05	160			
		6d^2^(^3^p)7s	^4^P	3/2	92	5	6d^2^(^1^D)7s	^2^D	13,361	12,847	1.676	1.6841	4.5e+05	210			
…																	
21,195.870	0.018	6d7s(^1^D)7p	^2^F°	5/2	41	29	6d7s(^3^D)7p	^4^P°	21,313	21,170	1.129	1.0394	23	10		TW,M	
21,232.31	0.05	6d7s(^3^D)7p	^4^D°	7/2	81	9	6d7s(^3^D)7p	^4^F°	20,537	20,288	1.391	1.3858	160	10		TW	T
		6d^2^(^1^S)7s	^2^S	1/2	81	5	6d^2^(^3^P)7s	^4^P	22,088	21,918	1.962	1.9806	8.e+04	2300			
…																	
35,870.009	0.022	6d^2^(^3^F)7p	^4^D°	7/2	32	24	6d^2^(^3^F)7p	^2^F°	35,313		1.249		8.1	10		TW,M	J
		6d7s(^3^D)7d	^4^G	5/2	62	21	6d7s(^3^D)7d	^2^F	36,102	36,150	0.723	0.6868	19.0	10			
		6d7s(^3^D)7d	^4^D	1/2	76	17	6d7s(^3^D)7d	^2^P	36,248		0.171		17.7	10			
		6d7s(^1^D)8s	^2^D	3/2	32	13	6d^2^(^1^D)8s	^2^D	36,322	36,218	0.850	0.8778	20.9	10			
43,394.52	0.10	Ac II (6p^6^7s^2 1^S_0_)	Limit													TW,R	

a The first percentage pertains to the configuration and term given in the columns “Configuration” and “Term”; the second one pertains to the configuration and term specified in the next two columns.

b In this work (TW), the energies were calculated in a least-squares fit with Cowan’s codes [21,22]. In the fitting, the calculated energies of Dzuba et al. [3] were used as “experimental” ones, where no experimental values are available.

c Landé g_*J*_-factors (dimensionless) calculated in this work (TW) and in Ref. [3].

d Radiative lifetimes calculated in this work (TW) and those calculated or measured in other works. Exponential notation is used in some of the *τ* values (e.g., “8.30e+09” means “8.30 × 10^9^”). For the values from this work, estimated percentage uncertainties are given in the next column. Reference values from other work are given with uncertainties specified in parentheses after the value in units of the last digit of the value. Their sources are specified as letter superscripts: D—Dzuba et al. [3] (theoretical); Z—Zhang et al. [2] (experimental).

e Code for references: F—Ferrer et al. [13]; M—Meggers et al. [9]; Z—Zhang et al. [2]; R—Roßnagel et al. [17]; TW—this work.

f Notes: J—the *J*-value (of Meggers et al. [9]) has been revised; N—a level newly identified in this work; R—revised identification; T—a new tentative identification of this work.

(Only a small portion of this table is given here for guidance to its content. The full version is available in machine-readable format in Table S2 of the Supplementary Online Materials, file Table S2.txt. The format of the supplementary table is slightly different. See footnotes to Table 1).

**Table 3.** Parameters of the least-squares fit for Ac I.

Parity	Configurations		Parameter	LSF^a^ (cm^−1^)	Δ^b^ (cm^−1^)	Group^c^	HFR^a^	LSF/HFR^a^
e	6d7s^2^		*E*_av_	3646.1	268		0.0	
e	6d7s^2^		ζ_6d_	855.0	126	6	1171.2	0.7300
e	6d7s7d		*E*_av_	40,623.1	459		33,548.7	1.2109
e	6d7s7d		ζ_6d_	991.7	146	6	1358.5	0.7300
e	6d7s7d		ζ_7d_	63.6	9	6	87.1	0.7302
e	6d7s7d		*F*^1^ (6d,7d)	0.0	fixed		0.0	
e	6d7s7d		*F*^2^(6d,7d)	2499.6	fixed		3570.9	0.7000
e	6d7s7d		*F*^3^(6d,7d)	0.0	fixed		0.0	
e	6d7s7d		*F*^4^(6d,7d)	1079.6	fixed		1542.3	0.7000
e	6d7s7d		*G*^2^(6d,7s)	13,953.6	fixed		19,933.7	0.7000
…								
e	6d^2^7s	6d^3^	$R_{d}^{2}$(6d7s,6d6d)	−13,229.4	1040	9	−20,926.0	0.6322
e	6d^2^8s	6d^3^	$R_{d}^{2}$(6d8s,6d6d)	−3654.5	287	9	−5780.6	0.6322
…								
o	6d7s7p		*E*_av_	23,249.1	239		16,467.7	1.4118
o	6d7s7p		ζ_6d_	1015.9	140	6	1272.5	0.7983
o	6d7s7p		ζ_7d_	2832.8	183	7	2195.9	1.2900
o	6d7s7p		*F*^1^(6d,7p)	0.0	fixed		0.0	
o	6d7s7p		*F*^2^(6d,7p)	8588.9	1001	5	14,362.0	0.5980
o	6d7s7p		*G*^2^(6d,7s)	14,850.0	2470	3	19,062.1	0.7790
o	6d7s7p		*G*^1^ (6d,7p)	6443.2	482	2	9766.1	0.6597
o	6d7s7p		*G*^2^(6d,7p)	0.0	fixed		0.0	
o	6d7s7p		*G*^3^(6d,7p)	2519.3	1221	10	6894.4	0.3654
o	6d7s7p		*G*^1^(7s,7p)	9196.1	774	4	20,369.4	0.4515
…								
o	6d7s7p	6d^2^7p	$R_{d}^{2}$(6d7s,6d6d)	−14,498.5	2703	8	−22,138.2	0.6549
o	6d7s8p	6d^2^8p	$R_{d}^{2}$(6d7s,6d6d)	−15,232.5	2840	8	−23,259.0	0.6549
…								

a Parameter values determined in the least-squares-fitted (LSF) and ab initio pseudo-relativistic Hartree–Fock (HFR) calculations and their ratio.

b Standard deviation of the fitted parameter. Parameters that were not varied in the fit are marked as “fixed”.

c Parameters in each numbered group were linked together with their ratio fixed at the HFR level.

(Only a small portion of this table is given here for guidance to its content. The full version is available in machine-readable format in Table S3 of the Supplementary Online Materials, file Table S3.txt).

**Table 4.** Spectral lines of Ac II.

*λ*_obs_^a^	*λ*_Ritz_^a^	Δ*λ*_O−R_^b^	*σ*_obs_^c^	*I*_obs_^d^	Lower Level		Upper Level		E_low_^e^	E_upp_^e^	A^f^	Acc.^g^	Type^h^	TP	Notes^j^
(Å)	(Å)	(Å)	(cm^−1^)	(arb.u.)	Configuration	Term_*J*_	Configuration	Term_*J*_	(cm^−1^)	(cm^−1^)	(s^−1^)			Ref.^i^	
2064.280(13)			48,427.6	14,000h											
2100.000(13)			47,603.9	27,000h											
2102.240(13)			47,553.2	3400h											
2261.749(6)	2261.7478(19)	0.001	44,199.89	12,000	7s^2^	^1^S_0_	7s7p	${\;}^{1}{\text{P}}_{1}^{{}^{\circ}}$	0.00	44,199.914	2.04e+08	C+		TW	
2307.500(13)			43,323.62	9800h											
2316.060(13)			43,163.51	3500											
2344.871(6)	2344.8721(20)	−0.001	42,633.21	4100	6d7s	^3^D_3_	5f6d	${\;}^{3}{\text{D}}_{3}^{{}^{\circ}}$	7426.489	50,059.68	8.e+06	E		TW	
2501.391(6)	2501.3942(17)	−0.003	39,965.71	2500	6d7s	^3^D_1_	5f6d	^1^D2	4739.631	44,705.290	8.e+06	E		TW	
…															
7567.652(9)	7567.647(7)	0.005	13,210.501	1800	6d^2^	^3^F_2_	6d7p	${\;}^{3}{\text{F}}_{2}^{{}^{\circ}}$	13,236.418	26,446.928	5.5e+06	C+		TW	
7617.421(9)	7617.410(7)	0.011	13,124.190	320	6d^2^	^3^F_4_	6d7p	${\;}^{3}{\text{F}}_{3}^{{}^{\circ}}$	16,756.847	29,881.055					
	7626.653(12)				6d7s	^1^D2	6d^2^	^1^D_2_	9087.517	22,199.428	3.2e+00	D+	M1 + E2	TW	
7886.822(9)	7886.821(7)	0.001	12,675.891	1200	6d^2^	^3^P_2_	6d7p	${\;}^{1}{\text{D}}_{2}^{{}^{\circ}}$	19,202.962	31,878.854	1.46e+06	C+		TW	
…															
	58,385.5(4)				6d^2^	^3^F_2_	6d^2^	^3^F_3_	13,236.418	14,949.173	1.217e−01	AA	M1	TW	
	60,203.7(6)				6d7s	^3^D_3_	6d7s	^1^D_2_	7426.489	9087.517	1.47e−02	C+	M1	TW	
	189,568(7)				6d7s	^3^D_1_	6d7s	^3^D_2_	4739.631	5267.147	3.11e−03	A+	M1	TW	

a Observed and Ritz wavelengths are given in standard air. Conversion between air and vacuum wavelengths was made with the five-parameter formula for the dispersion of air from Peck and Reeder [11]. All observed wavelengths are from the work of Meggers et al. [9].

b The difference between the observed and Ritz wavelengths. Blank for lines with unmeasured wavelength and for the lines that solely determine one of the energy levels of the transition.

c Transition wave number in vacuum.

d Observed intensity on an arbitrary scale, which is linear in terms of the energy flux under the line contour (see Section 6). The symbols after the numbers denote the character of the line: c—complex structure; h—hazy; l—shaded on the long-wavelength side; s—shaded on the short-wavelength side; *—the given intensity is shared by more than one transition.

e The optimized energies of the lower and upper levels of the transition. These values correspond to those given in Table 5.

f Transition probability. Exponential notation is used (e.g., “3.0e-01” means “3.0 × 10^−1^”).

g Accuracy of the transition probability. The code symbols for the accuracy are defined in the NIST ASD [8] (see https://physics.nist.gov/PhysRefData/ASD/Html/lineshelp.html#OUTACC, accessed on 25 March 2022).

h Transition type: blank—electric dipole; M1—magnetic dipole; E2—electric quadrupole; M1 + E2—mixed type (both types contribute more than 2% to the total *A* value given here).

i Code for references: R14—Roberts et al. [31]; TW—this work.

j Notes: N—a newly classified line; R—the previous classification (of Ref. [29]) has been revised; T—a new tentative identification.

(Only a small portion of this table is given here for guidance to its content. The full version is available in machine-readable format in Table S4 of the Supplementary Online Materials, file Table S4.txt. The format of the supplementary table is slightly different. See footnotes to Table 1).

**Table 5.** Energy levels of Ac II.

*E*_exp_	Unc.^a^	Configuration	Term	*J*	Leading Percentages^b^							*E*_calc_^c^	*g*_calc_^d^	τ^e^		Ref.^f^	Notes^g^
(cm^−1^)	(cm^−1^)											(cm^−1^)		(ns)	u%		
0.00	0.03	7s^2^	^1^S	0	95							0	0.000			TW,M,BW	
4739.631	0.018	6d7s	^3^D	1	99							4661	0.499	1.0e+23	52	TW,M,BW	
5267.147	0.015	6d7s	^3^D	2	86	11	6d7s	^1^D				5281	1.145	1.58e+11	19	TW,M,BW	
7426.489	0.016	6d7s	^3^D	3	99							7498	1.334	6.32e+09	1.7	TW,M,BW	
9087.517	0.014	6d7s	^1^D	2	68	16	6d^2^	^1^D	13	6d7s	^3^D	9087	1.015	1.61e+09	18	TW,M,BW	
13,236.418	0.000	6d^2^	^3^F	2	93							13,191	0.687	1.96e+09	22	TW,M,BW	
…																	
28,201.120	0.021	7s7p	^3^P°	2	89	7	6d7p	^3^P°				28,324	1.480	25.2	9	TW,M,BW	C
		6d^2^	^1^S	0	83	6	6d^2^	^3^P				29,025	0.000	4.e+04	760		
…																	
64,154.91	0.08	6d7d	^1^D	2	28	15	7p^2^	^1^D	13	7p^2^	^3^P	64,487	1.121	2.21	7	TW	T
64,285.02	0.05	6d7d	^3^F	4	72	18	6d7d	^3^G	6	6d7d	^1^G	64,218	1.194	3.07	5	TW,BW	C,J
64,332.11	0.06	5f7p	^3^D	3	47	17	6d7d	^3^D	17	5f7p	^1^F	64,316	1.225	3.01	5	TW	N
		5f7p	^1^D	2	13	27	6d7d	^1^D				64,660	1.146	2.32	6		
		6d7d	^3^P	1	64	29	6d7d	^3^S				65,242	1.635	2.2	12		
65,392.37	0.04	5f7p	^3^G	5	52	48	6d7d	^3^G				65,385	1.200	2.82	8	TW	RI
		6d7d	^1^G	4	66	11	6d7d	^3^ f				65,799	1.040	4.2	7		
		6d7d	^3^P	2	47	24	5f7p	^1^D				65,894	1.284	2.64	8		
		6d7d	^1^S	0	62	15	6d7d	^3^P				66,901	0.000	5.2	25		
68,692.14	0.06	7p^2^	^1^D	2	34	14	5f7p	^1^D	12	7p2	^3^P	68,668	1.089	1.40	6	TW	N
[94,800]^h^	250	Ac III (6p^6^7s^2^S_1/2_)	Limit													M74	

a Uncertainties are given for separations of the levels from the 6d^2 3^F_2_ level at 13,236.418 cm^−1^.

b The first percentage pertains to the configuration and term given in the columns “Configuration” and “Term”; the second and third ones pertain to the configuration and term specified next to the number.

c The energies calculated in this work in a least-squares fit with Cowan’s codes [21,22].

d Landé g_*J*_-factors (dimensionless) calculated in this work.

e Radiative lifetimes calculated in this work. Their estimated percentage uncertainties are given in the next column. Some of the lifetimes are given in exponential notation (e.g., “6.e+04” means “6. × 10^4^”).

f Code for references: BW—Blaise and Wyart [29]; M—Meggers et al. [9]; M74—Martin et al. [30]; TW—this work.

g Notes: C—the configuration and/or term assignment (of Blaise and Wyart [29]) have been revised in this work; J—the *J*-value (of Blaise and Wyart [29]) has been revised; N—a level newly identified in this work; RI—the level established by Meggers et al. [9], previously rejected by Blaise and Wyart [29], has been reinstated in this work; T—a new tentative identification of this work.

h A semiempirical value.

(Only a small portion of this table is given here for guidance to its content. The full version is available in machine-readable format in Table S5 of the Supplementary Online Materials, file Table S5.txt. The format of the supplementary table is slightly different. See footnotes to Table 1).

**Table 6.** Parameters of the least-squares fit for Ac II.

Parity	Configurations		Parameter	LSF^a^ (cm^−1^)	Δ^b^ (cm^−1^)	Gr.^c^	HFR^a^	Ratio^a^
e	7s^2^		*E*_av_	2627.6	171		0.0	
e	7s8s		*E*_av_	52, 346.6	132	3	48,226.9	1.0854
e	7s8s		G^0^(7s,8s)	833.9	127	9	1787.1	0.4666
e	7s9s		*E*_av_	70, 686.1	178	3	66,523.3	1.0626
e	7s9s		G^0^ (7s,9s)	274.6	42	9	588.4	0.4667
e	7s7d		*E*_av_	58, 920.4	262	6	54,156.8	1.0880
e	7s7d		ζ_7d_	209.8	8	10	250.9	0.8362
e	7s7d		G^2^(7s,7d)	1422.0	77	1	2076.6	0.6848
e	7s8d		*E*_av_	74, 175.2	330	6	69, 239.0	1.0713
e	7s8d		ζ_8d_	86.1	3	10	103.0	0.8359
e	7s8d		G^2^(7s,8d)	535.5	29	1	782.0	0.6848
…								
e	7s7d	6d8s	$R_{d}^{2}$(7s7d,6d8s)	3323.0	164	15	5862.0	0.5669
e	7s7d	6d8s	$R_{e}^{0}$(7s7d,6d8s)	1420.4	70	15	2505.7	0.5669
e	7s7d	5f7p	$R_{d}^{3}$ (7s7d,5f7p)	1174.0	58	15	2071.0	0.5669
e	7s7d	5f7p	$R_{e}^{1}$(7s7d,5f7p)	−1121.5	55	15	−1978.4	0.5669
e	6d7d	5f7p	$R_{d}^{1}$ (6d7d,5f7p)	−3663.2	181	15	−6462.0	0.5669
e	6d7d	5f7p	$R_{d}^{3}$(6d7d,5f7p)	−1039.9	51	15	−1834.4	0.5669
e	6d7d	5f7p	$R_{e}^{1}$(6d7d,5f7p)	462.8	23	15	816.4	0.5669
e	6d7d	5f7p	$R_{e}^{3}$(6d7d,5f7p)	−15.8	1	15	−27.9	0.5672
e	6d7d	7p^2^	$R_{d}^{1}$(6d7d,7p7p)	−3217.6	159	15	−5676.0	0.5669
e	6d7d	7p^2^	$R_{d}^{3}$(6d7d,7p7p)	−1547.2	76	15	−2729.3	0.5669
e	5f7p	7p^2^	$R_{d}^{2}$(5f7p,7p7p)	4234.6	209	15	7470.1	0.5669
o	7s7p		*E*_av_	29, 025.9	195		23,529.7	1.2336
o	7s7p		ζ_7p_	5144.4	172	5	3795.7	1.3553
o	7s7p		G^1^ (7s,7p)	15, 388.0	1104	4	25,295.3	0.6083
o	7s8p		*E*_av_	63, 502.9	fixed		58,060.7	1.0937
o	7s8p		ζ_8p_	1646.7	55	5	1215.0	1.3553
o	7s8p		G^1^ (7s,8p)	2166.8	155	4	3561.9	0.6083
…								
o	6d7p	5f7s	$R_{d}^{1}$(6d7p,5f7s)	−11,247.3	517	9	−19,768.3	0.5690
o	6d7p	5f7s	$R_{e}^{2}$(6d7p,5f7s)	−4957.7	703	7	−7495.6	0.6614
o	6d7p	5f8s	$R_{d}^{1}$(6d7p,5f8s)	−574.6	26	9	−1009.9	0.5690
o	6d7p	5f8s	$R_{e}^{2}$(6d7p,5f8s)	−1105.3	157	7	−1671.1	0.6614
o	6d7p	5f9s	$R_{d}^{1}$(6d7p,5f9s)	−194.8	9	9	−342.4	0.5689
o	6d7p	5f9s	$R_{e}^{2}$(6d7p,5f9s)	−553.4	78	7	−836.7	0.6614
o	6d7p	5f6d	$R_{d}^{1}$(6d7p,5f6d)	8235.1	378	9	14,474.0	0.5690
o	6d7p	5f6d	$R_{d}^{3}$(6d7p,5f6d)	4374.5	201	9	7688.6	0.5690
…								

a Parameter values determined in the least-squares-fitted (LSF) and ab initio pseudo-relativistic Hartree–Fock (HFR) calculations and their ratio.

b Standard deviation of the fitted parameter. Parameters that were not varied in the fit are marked as “fixed”.

c Parameters in each numbered group were linked together with their ratio fixed at the HFR level.

(Only a small portion of this table is given here for guidance to its content. The full version is available in machine-readable format in Table S6 of the Supplementary Online Materials, file Table S6.txt).

**Table 7.** Spectral lines of Ac III.

λ_obs_^a^	λ_Ritz_^a^	Δλ_O−R_^b^	σ_obs_^c^	*I*_obs_^d^	Lower Level		Upper Level		*E*_low_^e^	*E*_upp_^e^	*A* ^f^	Acc.^g^	Type^h^	TP
(Å)	(Å)	(Å)	(cm^−1^)	(arb.u.)	Configuration	Term_*J*_	Configuration	Term_*J*_	(cm^−1^)	(cm^−1^)	(s^−1^)			Ref.^i^
2626.440(6)	2626.439(5)	0.001	38,063.00	300,000 h	7s	^2^S_1/2_	7p	${\;}^{2}{\text{P}}_{3/2}^{{}^{\circ}}$	0.00	38,063.01	3.97e+08	A		R13,S07
2682.900(6)	2682.899(4)	0.001	37,262.03	23,000 h	6d	^2^D_3/2_	7p	${\;}^{2}{\text{P}}_{3/2}^{{}^{\circ}}$	800.97	38,063.01	2.89e+07	A		R13,S07
2952.550(6)	2952.551(5)	−0.001	33,859.13	230,000 h	6d	^2^D_5/2_	7p	${\;}^{2}{\text{P}}_{3/2}^{{}^{\circ}}$	4203.89	38,063.01	2.30e+08	A		R13,S07
3392.780(13)	3392.782(10)	−0.002	29,465.90	78,000 Dh	7s	^2^S_1/2_	7p	${\;}^{2}{\text{P}}_{1/2}^{{}^{\circ}}$	0.00	29,465.88	1.90e+08	A		R13,S07
3487.590(13)	3487.588(11)	0.002	28,664.89	99,000	6d	^2^D_3/2_	7p	${\;}^{2}{\text{P}}_{1/2}^{{}^{\circ}}$	800.97	29,465.88	1.58e+08	A		R13,S07
4413.090(13)	4413.093(11)	−0.003	22,653.50	34,000 h	6d	^2^D_3/2_	5f	${\;}^{2}{\text{F}}_{5/2}^{{}^{\circ}}$	800.97	23,454.45	1.85e+07	A		R13,S07
4569.870(13)	4569.870(13)		21,876.33	65,000 h	6d	^2^D_5/2_	5f	${\;}^{2}{\text{F}}_{7/2}^{{}^{\circ}}$	4203.89	26,080.22	2.11e+07	B+		R13,S07
5193.211(13)	5193.208(12)	0.003	19,250.55	710 h	6d	^2^D_5/2_	5f	${\;}^{2}{\text{F}}_{5/2}^{{}^{\circ}}$	4203.89	23,454.45	8.79e+05	B+		R13,S07
	23,787.5(5)				7s	^2^S_1/2_	6d	^2^D_5/2_	0.00	4203.89	3.748e–03	AA	E2	S17
	29,386.5(6)				6d	^2^D_3/2_	6d	^2^D_5/2_	800.97	4203.89	4.30e–01	A+	M1	S17
	124,849(14)				7s	^2^S_1/2_	6d	^2^D_3/2_	0.00	800.97	8.48e–07	AA	E2	S17

a Observed and Ritz wavelengths are given in standard air. Conversion between air and vacuum wavelengths was made with the five-parameter formula for the dispersion of air from Peck and Reeder [11]. All observed wavelengths are from the work of Meggers et al. [9].

b The difference between the observed and Ritz wavelengths. Blank for lines with unmeasured wavelength and for the lines that solely determine one of the energy levels of the transition.

c Transition wave number in vacuum.

d Observed intensity on an arbitrary scale, which is linear in terms of the energy flux under the line contour (see Section 6). The symbols after the numbers denote the character of the line: h—hazy; D—double line.

e The optimized energies of the lower and upper levels of the transition. These values correspond to those given in Table 8.

f Transition probability. Exponential notation is used (e.g., “3.97e+08” means “3.97 × 10^8^”).

g Accuracy of the transition probability. The code symbols for the accuracy are defined in the NIST ASD [8] (see https://physics.nist.gov/PhysRefData/ASD/Html/lineshelp.html#OUTACC, accessed on 25 March 2022).

h Transition type: blank—electric dipole; M1—magnetic dipole; E2—electric quadrupole.

i Code for transition probability references: R13—Roberts et al. [33]; S07—Safronova et al. [34]; S17—Safronova et al. [38].

**Table 8.** Energy levels of Ac III.

*E*_exp_	Unc.^a^	Configuration	Term	*J*	Perc.^b^	*g*_calc_^c^		[35]	*E*_calc_^d^ (cm^−1^)	[34]	τ^e^
(cm^−1^)	(cm^−1^)					TW	[39]		[33]		(ns)
0.00	0.09	7s	^2^S	1/2	99	2.002	2.005606	0	0	0	
800.97	0.06	6d	^2^D	3/2	99	0.800	0.798662	562	435	825	1.171(6)e15^S^
4203.89	0.00	6d	^2^D	5/2	99	1.200	1.200627	4040	3926	4041	2.305(34)e9^S^
23,454.45	0.04	5f	^2^F°	5/2	100	0.857		29,906	23,467	24,018	52(3)^R^
26,080.22	0.06	5f	^2^F°	7/2	100	1.143		32,063	26,112	26,420	48(3)^R^
29,465.88	0.10	7p	^2^P°	1/2	100	0.666		29,382	29,375	29,303	2.88(14)^R^
38,063.01	0.06	7p	^2^P°	3/2	100	1.334		37,987	38,136	37,816	1.53(8)^R^
[140,630]^f^	50	Ac IV (6p^6 1^S_0_)	Limit					140,590	141,221	140,442	

a Uncertainties are given for separations of the levels from the 6d ^2^D_5/2_ level at 4203.89 cm^−1^.

b Percentage of the configuration and term given in the columns “Configuration” and “Term” in the composition of the eigenvector (from the present Cowan-code calculation).

c Landé *g*_*J*_-factors (dimensionless) calculated in this work (TW) and in Gossel et al. [39].

d The calculated energies from Migdalek and Glowacz-Proszkiewicz [35], Roberts et al. [33], and Safronova et al. [34].

e Radiative lifetimes calculated in the works denoted by the superscripts: R—Roberts et al. [33]; S—Safronova et al. [38]. The first two values are given in the exponential notation, e.g., 1.171(6)e15 means (1.171 ± 0.006) × 10^15^.

f A semiempirical value derived in this work by isoelectronic interpolations (see the text of Section 5).

**Table 9.** Effective excitation temperatures (in eV) in the light sources used by Meggers et al. [9], determined from Boltzmann plots.

Spectrum	Ag Arc	Ag Spark	Cu Arc	Cu Spark	HC
Ac I	0.51	0.60	0.53	0.59	0.41
Ac II	0.63	0.77	0.56	0.77	0.64
Ac III	1.06	2.18	–	1.07	–

## Data Availability

Data are contained within the article and its Supplementary Materials.

## Abbreviations

The following abbreviations are used in this manuscript
ASD: Atomic Spectra Database
LTE: Local Thermodynamic Equilibrium
NIST: National Institute of Standards and Technology
